# Which Protein‐Based Dietary Supplements Most Effectively Enhance Fat‐Free Mass and Strength Gains in Healthy Adults Undergoing Resistance Training? A Network Meta‐Analysis

**DOI:** 10.1155/tsm2/5557511

**Published:** 2026-02-02

**Authors:** Marcos D. M. Drummond, Ronaldo A. D. Silva, Nelson Carvas Junior, Miércio S. Melo, Matheus H. L. Ferreira

**Affiliations:** ^1^ Laboratory of Nutrition and Sports Training, Federal University of Minas Gerais, Belo Horizonte, Minas Gerais, Brazil, ufmg.br; ^2^ State University of Paraná, Paranavaí, Paraná, Brazil; ^3^ Department of Evidence-Based Health, Federal University of São Paulo (UNIFESP), São Paulo, São Paulo, Brazil, unifesp.br

**Keywords:** body composition, dietary supplements, muscle hypertrophy, muscle strength, network meta-analysis as topic, resistance training

## Abstract

**Background:**

Various protein‐based dietary supplements are widely used by individuals engaged in strength training to optimize gains in muscle strength and fat‐free mass. However, gaps remain in the scientific literature regarding a comprehensive comparison—particularly the effectiveness of different types of supplemented proteins in healthy adults. This systematic review and network meta‐analysis aimed to compare the effectiveness of protein‐based dietary supplements, combined with strength training, on increasing muscle strength and fat‐free mass in healthy adults.

**Methods:**

A network meta‐analysis was conducted using randomized controlled trials evaluating different protein supplements combined with strength training. The outcomes assessed were muscle strength (primary) and fat‐free mass (secondary). The search was performed in PubMed, Scopus, and Embase up to May 2024, with no restrictions on language or publication date.

**Results:**

A total of 78 studies were included, comprising 4755 participants across two outcomes and involving 13 types of protein supplements, plus placebo and control groups. Compared to placebo, for strength, collagen was the most effective supplement (SMD = 0.41; 95% CI: 0.09 to 0.73; *p* = 0.0125; SUCRA 88.05%), followed by whey protein (SMD = 0.15; 95% CI: 0.03 to 0.27; *p* = 0.0145; SUCRA 64.34%). The other supplements showed no statistically significant differences compared to placebo (*p* > 0.05). For fat‐free mass, results were similar. Collagen showed a statistically superior effect (SMD = 0.94; 95% CI: 0.48 to 1.40; *p* < 0.0001; SUCRA 98.92%), followed by whey protein (SMD = 0.16; 95% CI: 0.05 to 0.28; *p* = 0.0051; SUCRA 60.23%). Other supplements showed no statistically significant differences (*p* > 0.05).

**Conclusion:**

Collagen and whey protein are the only protein supplements effective in enhancing strength training effects. Moreover, collagen shows a superior effect compared to whey protein for both outcomes.

## 1. Introduction

Resistance training (RT) constitutes the primary stimulus for increases in muscle strength and fat‐free mass (FFM) in healthy adults, whereas nutritional factors, particularly protein intake, exert an important supportive role [1, 2]. Adequate dietary protein may be obtained from whole‐food sources or supplements [3].

Recent evidence, however, demonstrates that physiological responses to isolated supplemental proteins can differ from those observed with whole‐food protein sources, even when total daily protein intake is matched [4–6]. Such differences stem from variations in amino acid composition, digestibility, absorption kinetics, and potential food‐matrix effects, all of which may modulate anabolic signaling and training‐induced adaptations [7, 8]. Consequently, comparative evaluation of commercially available protein supplements retains high clinical and practical relevance owing to their widespread adoption, convenience, and substantial influence on nutritional guidance and consumer behavior.

Protein‐based supplements differ markedly in origin and composition (e.g. whey, casein [CA], collagen [COL], egg, beef, soy, pea, rice, etc.) and therefore exhibit distinct capacities to support RT adaptations [9, 10]. Although conventional pairwise meta‐analyses have established that protein supplementation generally enhances gains in strength and FFM relative to placebo, especially when habitual dietary protein intake is suboptimal [11–13], these analyses have not generated a comparative hierarchy among protein sources.

Few network meta‐analyses (NMAs) have examined protein supplementation, and existing studies have either incorporated clinical populations, focused on older adults with sarcopenia, or failed to restrict inclusion to healthy individuals without chronic comorbidities [14, 15]. To date, no NMA has simultaneously compared all major supplemental protein types for effects on strength and FFM exclusively in healthy adults undergoing RT.

Methodological parallels exist with recent large‐scale NMAs in exercise science. For instance, Currier et al. employed a Bayesian NMA to rank resistance‐training prescriptions according to load, volume, and frequency, illustrating the utility of this approach for producing evidence‐based hierarchies across multiple interventions [16]. The present study applies an analogous analytical framework to the domain of nutritional supplementation.

The objective of this systematic review and NMA was therefore to compare the effectiveness of different protein‐based dietary supplements, in conjunction with RT, on muscle strength and FFM gains in healthy adults without chronic comorbidities. We hypothesized that animal‐derived proteins of high biological value would occupy the highest ranks, consistent with their complete essential amino acid profiles and well‐documented anabolic properties.

## 2. Materials and Methods

### 2.1. Protocol and Registration

This systematic review and NMA were conducted in accordance with the Preferred Reporting Items for Systematic Reviews and Meta‐Analyses incorporating Network Meta‐Analyses (PRISMA‐NMA) guidelines [17] and was prospectively registered in the PROSPERO database (ID: CRD42024510914).

Key methodological concepts underlying NMA—such as transitivity, consistency, indirect and mixed treatment comparisons, treatment ranking metrics (SUCRA), and network coherence—are detailed in Box 1 (Terminology: Reviews With Networks of Multiple Treatments), located in Section 1 of the Supporting Information.

### 2.2. Eligibility Criteria

We included randomized controlled trials (RCTs) that evaluated the effects of protein‐based dietary supplements, combined with RT, on muscle strength and FFM outcomes in healthy individuals without comorbidities.

The evaluated supplements were whey protein (WP), CA, soy protein (SP), milk protein (MP), COL, pea protein (PEAP), rice protein (RP), bovine colostrum (BC), beef protein (BP), peanut protein (PEANP), fish protein (FP), insect protein (IP), and lactoalbumin (LA). These protein sources were analyzed as distinct interventions rather than grouped, reflecting their unique amino acid profiles and their commercial availability as separate formulations (as some sources share nutritional similarities but are marketed and consumed differently, grouping was avoided to preserve analytical precision).

Placebo was treated as an active comparator, whereas control groups were analyzed separately when distinctly reported (noting that placebo often consisted of carbohydrate‐based interventions, which may have ergogenic effects in RT contexts, as addressed in the Limitations section).

The primary outcome was muscle strength, assessed using one‐repetition maximum (1RM), maximal voluntary isometric contraction (MVIC), or isokinetic tests. The secondary outcome was FFM, measured through dual‐energy X‐ray absorptiometry (DXA), bioelectrical impedance analysis (BIA), skinfolds, or equivalent methods. Baseline and postintervention values were extracted.

We excluded nonrandomized trials, observational studies, case reports, duplicates, unpublished academic works (such as theses and dissertations), narrative reviews, non‐peer‐reviewed articles, and trials evaluating protein combined with other ergogenic supplements.

Two potentially eligible studies could not be retrieved despite attempts to contact corresponding authors and were classified as “reports not retrieved” in accordance with PRISMA 2020 guidance.

### 2.3. Search

Searches were conducted in PubMed, Scopus, and Embase using controlled vocabulary (MeSH and Emtree) and free‐text terms related to the protein sources, outcomes of interest, and RT. In PubMed, MeSH terms were combined with filters for RCTs. In Scopus, indexing terms were searched in titles, abstracts, and keywords with RCT filters. In Embase, Emtree terms were applied with filters to exclude records already indexed in MEDLINE.

The search covered all records up to May 13, 2024, with no restrictions on language or publication date. Full search strategies for all databases are provided in Appendix Tables S1–S3 (Supporting Information 1—Section 2). Additionally, reference lists of eligible trials and previous systematic reviews were screened to identify studies not captured by the electronic search.

### 2.4. Information Sources

When full texts were not accessible, supplementary searches were conducted using repositories, open‐access platforms, and journal websites.

Corresponding authors were contacted when essential numerical data were missing or unclear.

### 2.5. Study Selection

Rayyan software [18] was used for duplicate removal and record management. Two reviewers (M.H.L.F. and M.S.M.) independently screened titles and abstracts, followed by full‐text assessment of potentially eligible studies. Discrepancies were resolved through consultation with a third reviewer (M.D.M.D.).

### 2.6. Data Extraction Process

Data extraction was performed independently by two reviewers (M.H.L.F. and M.S.M.) using a standardized Microsoft Excel sheet. Extracted information included: authorship, year, supplement type, outcomes, measurement methods, sample size, means and standard deviations (or convertible equivalents), participant characteristics (including habitual dietary protein intake when reported), and supplement dosage. Discrepancies were resolved by consensus with a third reviewer (M.D.M.D.).

### 2.7. Data Items

When data were reported as standard error, medians with ranges or interquartile intervals, or as confidence intervals, conversions to mean and SD followed Wan et al. [19] and Cochrane guidance [20].

For standardized mean difference (SMD) analyses, a correlation coefficient of 0.5 was assumed when not reported [20].

### 2.8. Network Geometry

Network geometry was depicted using nodes representing each supplement (scaled according to sample size) and edges representing direct comparisons (scaled by number of studies). Network connectivity and density were assessed visually and numerically to ensure that all treatments were connected, with placebo serving as the primary reference comparator.

### 2.9. Risk of Bias in Individual Studies and Certainty of the Evidence

Risk of bias was assessed using the Cochrane Risk of Bias 2.0 tool (RoB 2) [21], for each outcome. The six domains of the tool were independently evaluated by two reviewers (M.H.L.F. and M.S.M.), and any disagreements were resolved through consultation with a third reviewer (M.D.M.D.).

Certainty of the evidence was assessed using the CINeMA framework [22] based on GRADE principles [23], which consider within‐study bias, reporting bias, indirectness, imprecision, heterogeneity, and incoherence. The initial ratings generated by the CINeMA interface were reviewed by two independent assessors, and the final confidence in estimates was categorized as high, moderate, low, or very low according to GRADE criteria adapted for NMA.

### 2.10. Outcome Measures

Analyses followed a frequentist framework. Effect sizes were reported as SMD (Hedges’ g) with 95% confidence intervals, and statistical significance was set at *p* < 0.05.

Heterogeneity was assessed with Cochran’s Q test (*p* < 0.10 indicating significance) and the *I* ^2^ statistic, interpreted according to the thresholds proposed by Higgins et al. [24] and adopted in the Cochrane Handbook [20].

Effect sizes were interpreted according to Cohen’s criteria, in which SMD values of 0.2, 0.5, and ≥ 0.8 correspond to small, moderate, and large effects, respectively [25]. Although originally proposed for Cohen’s d, these thresholds are widely applied to Hedges’ g due to the conceptual equivalence between the two measures. As detailed by Borenstein et al. [26], Hedges’ g is a small‐sample corrected version of Cohen’s d, and this correction becomes negligible in large samples, allowing equivalent interpretation.

### 2.11. Planned Methods of Analysis

Random‐effects models using the inverse variance method were applied, and pairwise meta‐analyses were conducted when applicable.

To improve clinical interpretation, SMDs were converted to absolute units (kilograms) using representative standard deviations extracted from studies of similar populations [20, 26]. The adopted SDs were 14.76 kg for 1RM strength and 3.4 kg for FFM, derived from trials involving healthy adults undergoing RT.

When heterogeneity was significant, subgroup analyses were conducted based on follow‐up duration, participant age, and dosage. Habitual dietary protein intake and training history were considered as potential moderators but were not consistently reported across trials, precluding formal subgroup analysis or meta‐regression; this limitation is addressed in the Discussion section.

For studies reporting multiple supplement timing protocols, extraction followed this hierarchy: post‐exercise, pre‐exercise, before bedtime, and breakfast. For strength outcomes, lower‐limb results were prioritized.

### 2.12. SUCRA Ranking Analysis

Interventions were ranked using Surface Under the Cumulative Ranking Curve (SUCRA) values [27], ranging from 0% (worst) to 100% (best). In this study, SUCRA values are presented in decimal format, consistent with the statistical software output.

### 2.13. Assessment of Network Consistency (SIDE)

Local incoherence was assessed using the SIDE method [28], which compares direct and indirect estimates within each closed loop. A *p*‐value < 0.10 was interpreted as indicating significant incoherence; values between 0.05 and 0.10 were considered marginally incoherent depending on clinical context.

Additionally, the CINeMA framework [22] was used as a complementary tool to examine whether inconsistencies could be attributed to indirectness, such as differences in populations, interventions, or study contexts across direct and indirect comparisons.

### 2.14. Statistical Analysis

All analyses were conducted in R (version 4.4.1) [29] through RStudio [29]. The netmeta package [30] was used for NMAs, and the meta package [31] for pairwise comparisons. Auxiliary packages (tidyverse [32] and janitor [33]) were used to support data structuring. Rankograms and league tables were generated to visualize ranking distributions and comparative effects.

## 3. Results

**FIGURE 1:**
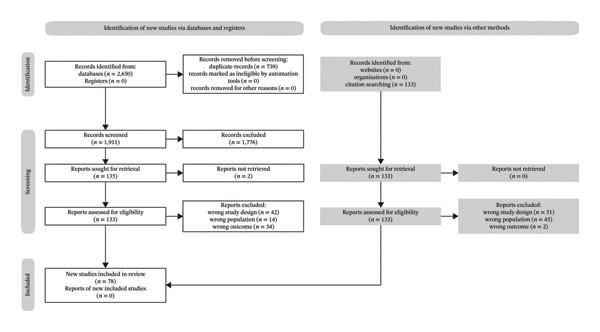
PRISMA flowchart. Legend: flowchart of study selection for inclusion in the meta‐analysis.

The systematic database search identified 2650 records, of which 739 were removed as duplicates. After title and/or abstract screening, 1776 records were excluded and 2 could not be retrieved, leaving 133 reports for full‐text assessment. Of these, 43 met eligibility criteria. An additional 35 studies were identified through citation searching and reference list screening, yielding a total of 78 included studies (Figure 1).

### 3.1. Network Geometry Summary

For the strength outcome (Figure 2(a)), 68 studies were included, totaling 92 comparisons among 13 different types of protein supplements, as well as placebo and control groups, across 24 study designs and involving 2401 participants. Of all studies included for the strength outcome, 49 used the 1RM method for assessment [34–56], [57–82]. Another 6 used MVIC [83–88], 5 used isokinetic dynamometry [89–93], 3 used 3RM [94–96], 2 used 5RM [97, 98], 1 used total load volume (maximum repetitions) [99], 1 used handgrip dynamometry [100], and 1 used the 8RM method [101].

Figure 2Network plots. (a) Strength; (b) fat‐free mass. Legend: Node size is proportional to the number of participants; edge thickness reflects the number of direct comparisons. Placebo served as the reference comparator.

**Figure:**
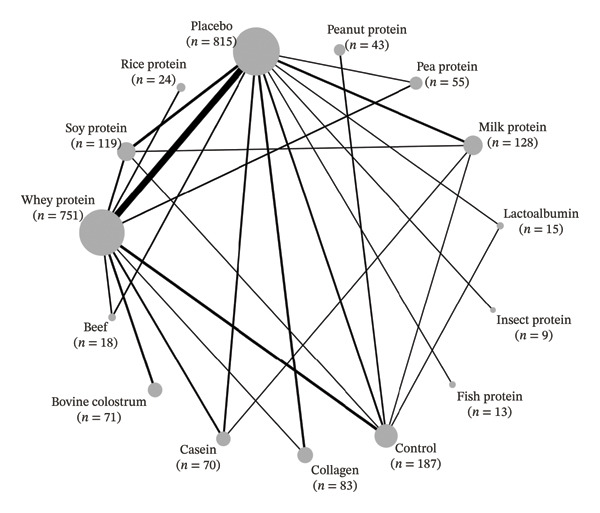
(a)

**Figure:**
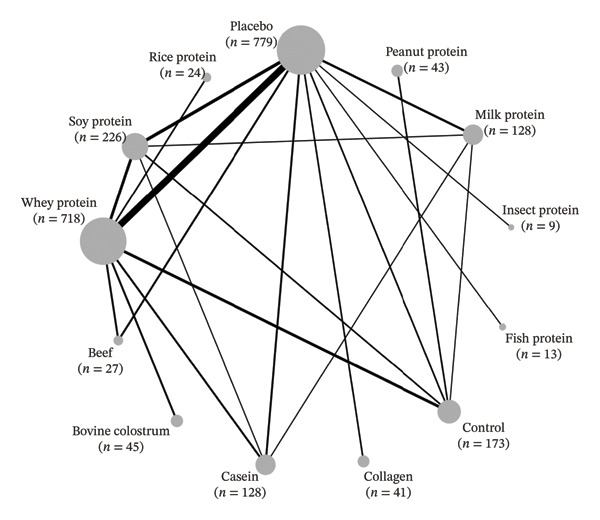
(b)

For the FFM outcome (Figure 2(b)), 63 studies were included, totaling 91 comparisons among 11 different protein supplements, in addition to placebo and control groups, distributed across 22 study designs and involving 2354 participants. Of these, 43 used the DXA method [34, 44, 45, 47–51, 54–56, 59–62, 64–67], [71–77, 79–83, 91, 93, 94, 96, 98, 99, 101–106], 13 used BIA [36, 41, 53, 63, 68–70, 78, 89, 90, 92, 100, 107], 3 used skinfold thickness [52, 85, 108], 2 used hydrostatic weighing [57, 109], 1 used magnetic resonance imaging [42], and 1 used air displacement plethysmography [110].

### 3.2. Study Characteristics

The samples from the included studies predominantly consisted of young adults, with mean ages ranging from 18 to 75 years. Most trials focused on individuals under 30 years of age. The mean age of participants varied according to the intervention and was reported separately for each group in all studies. Among the supplements investigated, the most frequently studied was WP, followed by SP, CA, MP, COL, PEAP, RP, BC, BP, FP, PEANP, IP, and LA. Placebo was used as the primary comparator in the analyses, while the control group was included separately when distinctly reported in the original studies.

Based on these studies, a detailed assessment of the tested interventions was conducted, including administered doses, weekly training frequency, and follow‐up duration. Detailed characteristics of the included studies, including participant demographics, interventions, sample sizes, and follow‐up duration, are presented in Appendix Tables S4 and S5 (Supporting Information 1—Section 3).

### 3.3. Risk of Bias in the Included Studies

In the strength outcome, the risk of bias assessment showed the following results: Domain 1 (bias arising from the randomization process) indicated that 33.8% of studies had low risk, 57.4% had some concerns, and 8.8% had high risk. Domain 2 (bias due to deviations from intended interventions) showed 98.5% of studies with low risk and only 1.5% with some concerns. For Domain 3 (bias due to missing outcome data), 72.1% were classified as low risk, 23.5% as having some concerns, and 4.4% as high risk. Domain 4 (bias in outcome measurement) showed 98.5% with low risk and 1.5% with some concerns. In contrast, Domain 5 (bias in the selection of reported results) demonstrated greater vulnerability, with 75% of studies classified as having some concerns and only 25% with low risk. Finally, the overall risk of bias (Domain 6) was considered low in 73.5% of studies, while 13.2% had some concerns and 13.2% were classified as high risk.

**FIGURE 3:**
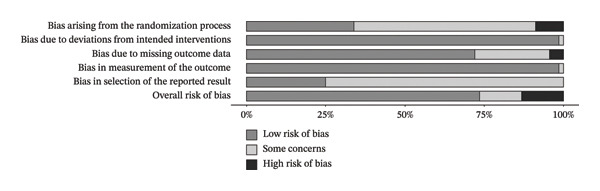
Risk of bias for the strength outcome. Bars represent the proportion of studies judged as low risk of bias (dark gray), some concerns (light gray), or high risk of bias (black) for each domain and overall.

**Figure 4:**
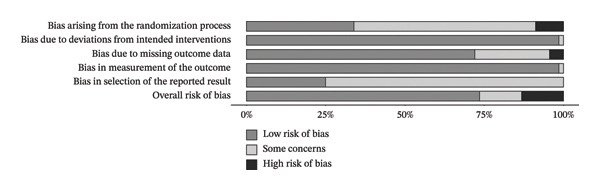
Risk of bias to FFM outcome. Legend: Bars represent the proportion of studies judged as low risk of bias (dark gray), some concerns (light gray), or high risk of bias (black) for each domain and overall.

In the FFM outcome, the assessment indicated that Domain 1 (bias arising from the randomization process) showed low risk of bias in 34.9% of studies, while 55.6% had some concerns and 9.5% were classified as high risk. Domain 2 (bias due to deviations from intended interventions) showed low risk in 98.4% of studies, with only 1.6% presenting some concerns. Domain 3 (bias due to missing outcome data) showed low risk in 66.7%, some concerns in 28.6%, and high risk in 4.8%. Regarding Domain 4 (bias in outcome measurement), 98.4% of studies were classified as low risk, with only 1.6% presenting some concerns. In Domain 5 (bias in selection of the reported result), most studies (73%) had some concerns, and 27% were classified as low risk. Finally, Domain 6 (overall risk of bias) indicated low risk in 71.4% of studies, while 14.3% had some concerns and 14.3% were classified as high risk. The risk of bias for the included studies is presented in Figures 3 and 4. Detailed risk of bias assessments for each individual study is provided in Appendix Figures S1 and S2 (Supporting Information 1 – Section 4).

### 3.4. Individual Study Results

A summary of the main findings from each study is provided in Appendix Table S6 (Supporting Information 1—Section 5).

## 4. Summary of Results

### 4.1. Strength

In the strength outcome, COL emerged as the most effective supplement among those investigated. It demonstrated statistical superiority over CA (SMD = 0.55; 95% CI: 0.10 to 1.00; *p* = 0.0171), MP (SMD = 0.53; 95% CI: 0.12 to 0.93; *p* = 0.0115), and placebo (SMD = 0.41; 95% CI: 0.09 to 0.73; *p* = 0.0125), with a significant increase in muscle strength. WP also showed a significant improvement compared to placebo (SMD = 0.15; 95% CI: 0.03 to 0.27; *p* = 0.0145), being, along with COL, the only supplement with statistical evidence of benefit for this outcome.

The SUCRA analysis revealed that COL had the highest probability of being the most effective intervention (SUCRA = 88.05%), followed by BC (76.81%), BP (66.66%), WP (64.34%), PEAP (62.89%), and LA (62.02%), all with values above 60%, indicating potential benefit over placebo. In contrast, interventions such as MP (23.59%) and CA (23.46%) showed the lowest SUCRA values, suggesting a low probability of being the most effective options.

Global network heterogeneity was considered negligible, with *τ* ^2^ = 0.0077, *τ* = 0.0877, and *I* ^2^ = 5.6% (95% CI: 0.0%–29.9%), indicating low variability among studies. The within‐design heterogeneity analysis did not reveal statistically significant differences (*Q* = 59.36; df = 48; *p* = 0.13), and no significant inconsistency was observed between different network designs (between designs: *Q* = 10.53; df = 18; *p* = 0.91). The global incoherence test also showed no relevant discrepancies between direct and indirect comparisons (*Q* = 69.90; df = 66; *p* = 0.35). These results demonstrate good internal consistency of the network, suggesting that the estimated effects are reliable and that the network structure is methodologically robust.

### 4.2. FFM

COL stood out as the most effective supplement among those investigated. Compared to placebo, COL showed a statistically significant increase in FFM (SMD = 0.94; 95% CI: 0.48 to 1.40; *p* < 0.0001). Additionally, it demonstrated statistical superiority over a wide range of supplements, including BC (SMD = 0.70; 95% CI: 0.08 to 1.32; *p* = 0.027), CA (SMD = 0.87; 95% CI: 0.35 to 1.39; *p* = 0.0011), FP (SMD = 0.96; 95% CI: 0.05 to 1.87; *p* = 0.038), MP (SMD = 0.83; 95% CI: 0.32 to 1.35; *p* = 0.0015), PEANP (SMD = 0.91; 95% CI: 0.24 to 1.58; *p* = 0.0077), RP (SMD = 0.88; 95% CI: 0.15 to 1.62; *p* = 0.019), SP (SMD = 0.85; 95% CI: 0.36 to 1.35; *p* = 0.0007), and the control group (SMD = 0.87; 95% CI: 0.35 to 1.39; *p* = 0.0010). Additionally, COL was significantly superior to WP (SMD = 0.78; 95% CI: 0.31 to 1.25; *p* = 0.0012). Besides COL, WP supplement demonstrated a statistically significant increase in FFM compared to placebo (SMD = 0.16; 95% CI: 0.05 to 0.28; *p* = 0.0051).

The SUCRA ranking indicated that COL emerged as the intervention with the highest probability of being the most effective (SUCRA = 98.92%), followed by BP (77.00%) and BC (64.12%), demonstrating superior performance compared to the other supplements. WP also showed a favorable ranking (60.23%), surpassing placebo (39.7%) and other protein sources with lower probabilities of efficacy, such as MP (48.57%), SP (43.22%), and RP (42.66%). The lowest SUCRA values were observed for FP (34.34%), PEANP (37.30%), IP (38.35%), and CA (40.72%), indicating that these interventions are less likely to be among the most effective.

Based on the results of the heterogeneity and inconsistency analysis, the network demonstrated excellent consistency and homogeneity among the included studies. Total heterogeneity was considered low, with *τ* ^2^ = 0, *τ* = 0, and *I* ^2^ = 0% (95% CI: 0.0%–29.3%). The within‐design heterogeneity test did not indicate statistically significant variations among studies (*Q* = 27.51; df = 48; *p* = 0.9923), and no significant inconsistency was observed between different network designs (between designs: *Q* = 21.10; df = 17; *p* = 0.2219). Additionally, the global incoherence test also revealed no relevant discrepancies between direct and indirect network data (*Q* = 48.61; df = 65; *p* = 0.9357). These results demonstrate good internal consistency of the network, suggesting that the estimated effects are reliable and that the network structure is methodologically robust. The corresponding NMA estimates are presented in Figure 5.

Figure 5Forest plots from the network meta‐analysis using a random‐effects model. The placebo group was used as the reference comparator. (a) Primary outcome: muscle strength; (b) secondary outcome: fat‐free mass. Legend: Results are presented as standardized mean differences (SMD) with 95% confidence intervals (95% CI) and SUCRA values, which represent the probability of each treatment being among the most effective.

**Figure:**
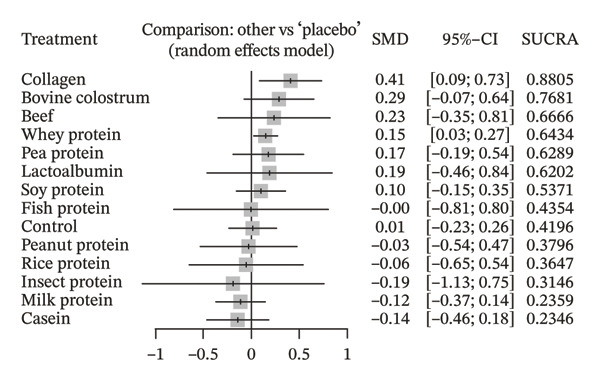
(a)

**Figure:**
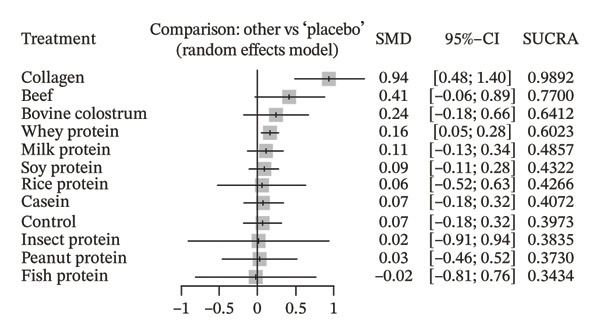
(b)

Table 1 presents a summary of the comparisons between protein supplements and placebo that showed statistically significant differences in the evaluated outcomes. The SMD, 95% CI, *p* value, the approximate estimate converted to real units (mean value, best‐case scenario, and worst‐case scenario), and the effect size classification are described.

**Table 1:** Comparisons of supplements that showed statistically significant differences compared to the placebo group for the strength and FFM outcomes.

Comparison	Outcome	SMD (95% CI)	*p*‐value	Approximate value (real unit)	Effect size
Collagen vs Placebo	Muscle strength	0.41 [0.09; 0.73]	0.0125	6.05 kg [1.33; 10.77] kg	Small‐Moderate
Whey protein vs Placebo	Muscle strength	0.15 [0.03; 0.27]	0.0145	2.21 kg [0.44; 3.99] kg	Small
Collagen vs Placebo	Fat‐free mass	0.94 [0.48; 1.40]	< 0.0001	3.20 kg [1.63; 4.76] kg	Large
Whey protein vs Placebo	Fat‐free mass	0.16 [0.05; 0.28]	0.0051	0.54 kg [0.17; 0.59] kg	Small

*Note:* Legend: Comparisons showing statistically significant differences between supplements and placebo for the outcomes of muscle strength and fat‐free mass. Values are expressed as standardized mean differences (SMD), confidence intervals (95% CI), *p*‐values, approximate conversion to real units (kilograms), and effect size classification.

All SMD values and their respective 95% confidence intervals for all comparisons are presented in Appendix Tables S7 and S8, organized in a league table format for both the primary outcome (strength) and the secondary outcome (FFM) (Supporting Information 1—Section 6).

In the present study, the detailed results of the treatment ranking analyses were also illustrated using rankograms, which provide a clear view of the probabilities associated with each ranking position for each treatment. To enhance clarity and data transparency, the complete rankograms are provided in Appendix Figures S3 and S4 (Supporting Information 1—Section 7).

### 4.3. Results of the Certainty of Evidence Assessment

The results of the certainty of evidence assessment, conducted using the CINeMA platform, indicated that most comparisons, for both outcomes, were rated as having moderate confidence, with within‐study bias being the main reason for downgrading. Comparisons with high confidence were less frequent. On the other hand, some comparisons were rated as low or very low confidence, especially those with a limited number of direct studies, wide confidence intervals (imprecision), the presence of indirectness, or considerable heterogeneity. Comparisons based on a single study with imprecise effect estimates were particularly vulnerable to downgrading the certainty of the evidence. Additionally, evidence related to certain proteins such as fish, insect, and PEANP proved to be especially fragile, often receiving a “very low” rating due to the combination of bias, imprecision, and indirectness. These findings highlight the importance of evaluating not only the magnitude of the estimated effects but also the methodological robustness and consistency of the contributing evidence when interpreting the results of an NMA. The full CINeMA assessment for each outcome is provided in Appendix Figures S5 and S6 (Supporting Information 1—Section 8).

### 4.4. Results of Additional Analyses

The local incoherence analysis which assesses whether there is inconsistency between results obtained from direct comparisons (between two supplements evaluated within the same study) and indirect comparisons (comparisons mediated by a third treatment) indicated that, in most comparisons, there was good agreement between estimates, suggesting network coherence (*p* ≥ 0.10). However, three comparisons showed signs of incoherence. For the FFM outcome, the comparison between SP and WP showed statistically significant incoherence (*p* = 0.0323), while the comparison between the control group and SP had a *p*‐value of 0.0906, being classified as marginally incoherent. For the strength outcome, the comparison between the control group and SP also showed a *p*‐value of 0.0583, representing a third case of marginal incoherence. All other comparisons demonstrated consistency between direct and indirect effects.

The statistical incoherence observed between SP and WP (*p* = 0.0323) was investigated through a sensitivity analysis, excluding studies classified as having a high risk of bias. The incoherence persisted, suggesting that the observed discrepancy is not solely due to methodological limitations. According to the complementary assessment using the CINeMA tool, the incoherence may be related to issues of indirectness, meaning differences in populations, interventions, or study contexts among the included trials. The complete results of the network coherence assessment are presented in Appendix Figures S7 and S8 (Supporting Information 1—Section 9). All pairwise meta‐analyses are presented in Appendix Figures S9–S18 (Supporting Information 1—Section 10).

## 5. Discussion

The present NMA investigated the comparative effects of protein‐based dietary supplements in healthy adults undergoing RT, focusing on muscle strength and FFM. Our initial hypothesis—that high‐biological‐value, animal‐derived proteins would be most effective—was partially supported, as only COL and WP showed statistically significant improvements over placebo for both outcomes, with COL ranking higher in the treatment hierarchy (SUCRA values of 94.5% vs. 78.2% for strength and 96.8% vs. 74.6% for FFM).

These findings must be interpreted with caution, as they are driven largely by indirect evidence in the network, given the limited number of direct head‐to‐head trials (only one comparing COL vs. WP). The low heterogeneity (*I* ^2^ ≈ 0%–5.6%) and nonsignificant inconsistency support the robustness of comparisons, but rankings for COL may be less stable due to fewer trials (comparable to other minor supplements but far below WP). To contextualize practical significance, SMD conversions indicate modest but meaningful absolute gains (e.g., ∼6.1 kg strength and ∼1.8 kg FFM with COL vs. placebo over 8–16 weeks), aligning with real‐world RT improvements. These differences, though statistically robust, are small and should be balanced against cost, sustainability, and individual factors in recommendations.

Our methodological approach aligns with prior large‐scale NMAs in exercise science. For instance, Liao et al. [14] ranked RT prescriptions (load, sets, frequency) using NMA, demonstrating its utility for multi‐intervention comparisons. Here, we applied the same framework to protein supplements, highlighting COL and WP as top‐ranked strategies. This parallel is methodological, not mechanistic, emphasizing NMA’s role in evidence‐based guidance.

The results of this study highlight the positive effects of COL supplementation compared to placebo, particularly for improvements in muscle strength and FFM. Although most systematic reviews with meta‐analyses to date have focused on the effects of COL on skin health [111–113], our findings are consistent with a recent systematic review and meta‐analysis that demonstrated significant increases in FFM, along with reductions in fat mass, serum LDL cholesterol, and systolic blood pressure, without affecting glycemic markers [114]. These results are also consistent with a systematic review with NMA that evaluated different types of proteins and concluded, with moderate certainty of evidence, that COL is effective in increasing both FFM and appendicular lean mass [13]. Additionally, a systematic review with meta‐analysis conducted by Bischof et al. [115] reinforces these findings by showing that daily COL supplementation significantly increased FFM and muscle strength, with moderate and low certainty of evidence, respectively.

Despite having low biological value and a low content of branched‐chain amino acids (BCAAs), COL is rapidly absorbed in the small intestine, which may favor recovery after physical exercise—even though the timing of protein intake during or around a training session is not associated with improvements in strength or muscle hypertrophy [116]. A double‐blind RCT demonstrated that ingesting 30 g of COL before high‐intensity resistance exercise increased whole‐body COL synthesis [117]. Additionally, the levels of arginine and glycine present in this supplement—amino acids that serve as fundamental substrates in the endogenous creatine synthesis pathway—may enhance creatine production in the body, which is associated with increases in lean mass [118] and improvements in muscle function [119]. COL peptides have been identified as positive promoters of microcirculation [120]. The increase in microvascular perfusion results in greater amino acid supply and can be associated with enhanced anabolic responses following protein supplementation [120]. Thus, COL supplementation has a possible additional potential beneficial effect in promoting muscle growth when compared to other protein sources. It is also speculated that the reduction in joint pain may have occurred with the use of this supplement, consequently improving RT performance and the evaluated outcomes [121, 122]. A systematic review concluded that COL is beneficial for improving functionality and reducing joint pain, as well as enhancing body composition, muscle strength, and recovery [120].

The effect of enhancing the increase in FFM may also occur due to the abundant presence of COL in the structure of skeletal muscle and adjacent tissues [123]. This indicates that specificity, determined by the structural similarity of a protein source, can be a factor that overrides or renders unnecessary its high biological value. Therefore, COL supplementation may not be rich in BCAAs, but it provides the necessary amino acids for the synthesis of an abundant protein in the skeletal muscle structure, with a specific amino acid profile, ingested simultaneously. Further studies are needed to corroborate such a hypothesis.

WP supplementation was also superior to placebo for both muscle strength and FFM gains. These results are consistent with the existing literature, which supports WP supplementation in conjunction with RT sessions [124], showing increases in lean mass and muscle strength in healthy young individuals [125], men [126], and sarcopenic older adults [127]. In addition to the benefits related to the outcomes evaluated in the present study, WP may improve glycemic control in adults by reducing fasting insulin, fasting glucose levels, and insulin resistance as assessed by the Homeostasis Model Assessment of Insulin Resistance (HOMA‐IR) [128]. In patients with metabolic syndrome and related conditions, WP has shown beneficial effects on several indicators of glycemic control and lipid parameters [129]. The likely mechanism of action involves stimulation of insulin and incretin hormone (GIP and GLP‐1) secretion, appetite suppression, and delayed gastric emptying. In healthy individuals, the glucose‐lowering mechanisms associated with WP intake are the same as those observed in patients with impaired fasting glucose or diabetes mellitus [130]. Due to its high BCAA content, especially leucine, WP appears to promote muscle protein synthesis when ingested after exercise and during the recovery period between training sessions, provided it is consumed in sufficient quantities [89]. Although most studies have used concentrated WP, current literature suggests no differences in body composition among physically active individuals when comparing its concentrated, hydrolyzed, and isolated forms [131].

The ineffectiveness of the plant‐based proteins investigated in the present study may be attributed to their amino acid profile, characterized by a low concentration of key amino acids for human protein synthesis [132], such as leucine. However, the results of this study suggest that protein quality alone may not guarantee a supplement’s efficacy as a potentiator of RT effects, as other high biological value, animal‐derived proteins were not effective. Therefore, additional factors may influence the effects of supplementation when combined with RT, such as protein digestibility and absorption rate [132], as well as ancillary effects beyond direct protein synthesis, including enhanced microcirculation, joint strengthening [120, 124], and amino acids profile specificity. This reasoning may also help explain the superiority of COL supplementation over WP.

Additionally, the quality and total quantity of daily protein intake (from both diet and supplementation), ideally close to 1.6 g/kg, are factors that directly influence the efficacy of supplementation for both strength gains and FFM gains [10]. Supplementation alone may not be sufficient, nor may it even be necessary, to stimulate positive physiological adaptations for these analyzed outcomes [116]. Moreover, factors such as effective training load, strength training protocol, fitness level, and age can also directly influence FFM and strength gains [1], which, in turn, directly affect the efficacy of supplementation. However, the results of the present study demonstrated that COL and WP protein‐based supplements can effectively enhance the RT gains in strength and FFM. Thus, these effects can be indirect, attributed to the supplements’ aid in reaching the required quantity and quality of dietary protein intake, thereby acting as an effective nutritional aid. It is not clear what the possible direct ergogenic effect of these protein‐based supplements is, particularly concerning strength gains.

The superiority of the placebo group over some interventions, as indicated by the SUCRA values, may also be attributed to the ergogenic effects of carbohydrates on RT performance, given that most studies included in this review used carbohydrates as the placebo supplement. Meta‐regression analyses have demonstrated that the number of sets performed to maximal effort is an important moderator of the effect size magnitude of carbohydrate intake on RT performance [133].

### 5.1. Limitations

The present review has several limitations. The number of studies varied considerably across supplement categories, which reduces the precision of estimates for supplements informed by few trials. Much of the comparative evidence, especially for COL relative to other protein sources, is based on indirect pathways because only one trial directly compared COL with WP. This analytical framework is an inherent characteristic of NMA, which synthesizes direct and indirect evidence to generate coherent estimates of comparative effectiveness, as described by Salanti et al. [27]. The methodological foundation for this approach is further reinforced by empirical demonstrations showing that living NMAs can provide more reliable and timely estimates than traditional pairwise meta‐analyses, as reported by Nikolakopoulou et al. [134]. Variability in RT protocols, participant characteristics, and intervention duration may also influence effect sizes. Important moderators such as habitual dietary protein intake, training history, and total energy intake were rarely reported and therefore could not be examined. Even so, the use of random effects models and the very low heterogeneity observed in the analyses suggest that unreported variability did not meaningfully distort comparative estimates. At the same time, this variability may enhance ecological validity because real‐world RT and supplementation practices rarely involve strict dietary or training control.

## 6. Conclusion

In conclusion, COL and WP were the only supplements that consistently improved the effects of RT on muscle strength and FFM. COL achieved higher rankings than WP for both outcomes; however, these estimates were informed by relatively few studies and were largely derived from indirect comparisons. Therefore, although the probability rankings favor COL, these findings should be interpreted with caution. Additional high‐quality randomized trials, particularly direct head‐to‐head comparisons, are needed to confirm the comparative effectiveness of these protein‐based supplements.

### 6.1. Perspectives

The findings of this systematic review with NMA challenge several longstanding assumptions in the literature. First, despite the common belief that COL is not effective for enhancing strength or increasing FFM, the present analysis identified statistically significant effects for both outcomes. Second, the results do not fully support the traditional hierarchy that assumes universal superiority of animal‐derived proteins over plant‐based sources, nor the notion that protein supplementation offers minimal ergogenic value when combined with RT. Similar patterns have been reported in recent NMAs, suggesting that these conclusions warrant renewed examination.

Nevertheless, these observations should be interpreted cautiously because evidence for several supplement comparisons, particularly those involving COL, relies heavily on indirect estimates informed by relatively few trials. Additional rigorously designed studies, including direct head‐to‐head comparisons with larger samples and standardized training protocols, are needed to confirm or refute the relative advantages suggested by the present network.

From a practical perspective, this NMA provides preliminary evidence‐based guidance that may support decision‐making for professionals involved in nutritional prescription and RT programming. Just as Currier et al. [16] developed structured evidence‐based rankings for training variables, the present NMA offers an analogous framework for considering protein supplement options. Together, these lines of research highlight the complementary roles of training design and dietary strategies in optimizing strength and hypertrophy adaptations in healthy adults.

## Author Contributions

Conceptualization: Marcos D. M. Drummond and Matheus H. L. Ferreira.

Methodology: Marcos D. M. Drummond, Miércio S. Melo, and Matheus H. L. Ferreira.

Data curation: Miércio S. Melo and Matheus H. L. Ferreira.

Formal analysis: Matheus H. L. Ferreira, Nelson Carvas Junior.

Writing–original draft: Miércio S. Melo, Matheus H. L. Ferreira, Ronaldo A. D. Silva, and Nelson Carvas Junior.

Writing–review and editing: Marcos D. M. Drummond, Matheus H. L. Ferreira, Ronaldo A. D. Silva, and Nelson Carvas Junior.

Supervision: Marcos D. M. Drummond.

## Funding

This research did not receive any specific grant from funding agencies in the public, commercial, or not‐for‐profit sectors.

## Disclosure

All authors approved the final manuscript.

## Conflicts of Interest

The authors declare no conflicts of interest.

## Supporting Information

The supporting information accompanying this article includes the following sections:

2. Search Strategy: Detailed search strategies used for PubMed, Scopus, and Embase (Tables S1–S3).

3. Study Characteristics: Summary of the included studies with information on interventions, sample sizes, participant ages, doses, frequency, and follow‐up durations (Tables S4–S5).

4. Risk of Bias Assessment: Traffic plots illustrating the risk of bias across the included studies for both strength and fat‐free mass outcomes (Figures S1 and S2).

5. Individual Study Results: Results extracted from each included study (Table S6).

6. League Tables: Relative effects for all pairwise comparisons for both outcomes (Tables S7 and S8).

7. Rankograms: Ranking probabilities of each intervention for both outcomes (Figures S3 and S4).

8. CINeMA Assessment: Confidence ratings in the network meta‐analysis findings using the CINeMA framework (Figures S5 and S6).

9. Network Coherence Assessment: Graphical assessments of network coherence for both outcomes (Figures S7 and S8).

10. Pairwise Meta‐Analyses: Forest plots of traditional pairwise meta‐analyses comparing individual supplements against placebo (Figures S9–S18).

Supporting Description

The supporting information includes

i. Box 1 (Section 1): Terminology: Reviews With Networks of Multiple Treatments, providing key methodological definitions related to network meta‐analysis;

ii. All appendix figures and tables (Sections 2–11), including detailed search strategies, study characteristics, risk of bias assessments, league tables, rankograms, CINeMA evaluations, coherence analyses, and pairwise meta‐analyses.

## Supporting information


**Supporting Information** Additional supporting information can be found online in the Supporting Information section.

## Data Availability

The data that support the findings of this study are available from the corresponding author upon reasonable request.
