# Supplementary material for: Which Protein‐Based Dietary Supplements Most Effectively Enhance Fat‐Free Mass and Strength Gains in Healthy Adults Undergoing Resistance Training? A Network Meta‐Analysis
Source: Transl Sports Med. 2026 Feb 2;2026:5557511. doi: 10.1155/tsm2/5557511 (PMC12862422; doi:10.1155/tsm2/5557511)

**SUPPLEMENTARY MATERIAL**

**Section 1 – Terminology 3**

Box 1. Terminology: Reviews With Networks of Multiple Treatments 3

**Section 2 – Search Strategy 4**

2.1 Detailed Search Strategy 4

2.1.1 Table S1: Search Strategy in PubMed 4

2.1.2 Table S2: Search Strategy in Scopus 4

2.1.3 Table S3: Search Strategy in Embase 5

**Section 3 – Study Characteristics 6**

3.1 Table S4: Characteristics of the Included Studies 7

3.2 Table S5: Included Studies in the Review 11

**Section 4 – Risk of Bias Assessment 16**

4.1 Figure S1: Traffic Plots – Risk of Bias – Strength Outcome 16

4.2 Figure S2: Traffic Plots – Risk of Bias – Fat-Free Mass Outcome 17

**Section 5 – Individual Study Results 18**

5.1 Table S6: Individual Results of the Included Studies 18

**Section 6 – League Tables 24**

6.1 Table S7: League Table – Strength Outcome 24

6.2 Table S8: League Table – Fat-Free Mass Outcome 26

**Section 7 – Rankograms 27**

7.1 Figure S3: Rankogram – Strength Outcome 27

7.2 Figure S4: Rankogram – Fat-Free Mass Outcome 28

**Section 8 – CINeMA Assessment 29**

8.1 Figure S5: CINeMA for the Strength Outcome 29

8.2 Figure S6: CINeMA for the Fat-Free Mass Outcome 33

**Section 9 – Network Coherence Assessment 36**

9.1 Figure S7: Network Coherence for the Strength Outcome 36

9.2 Figure S8: Network Coherence for the Fat-Free Mass Outcome 37

**Section 10 – Pairwise Meta-Analysis 38**

10.1 Figure S9: Strength Outcome – Whey Protein vs Placebo 38

10.2 Figure S10: Strength Outcome – Collagen vs Placebo 38

10.3 Figure S11: Strength Outcome – Soy Protein vs Placebo 39

10.4 Figure S12: Strength Outcome – Casein vs Placebo 39

10.5 Figure S13: Strength Outcome – Beef vs Placebo 39

10.6 Figure S14: Strength Outcome – Milk Protein vs Placebo 39

10.7 Figure S15: Fat-Free Mass Outcome – Whey Protein vs Placebo 40

10.8 Figure S16: Fat-Free Mass Outcome – Collagen vs Placebo 40

10.9 Figure S17: Fat-Free Mass Outcome – Beef vs Placebo 40

10.10 Figure S18: Fat-Free Mass Outcome – Milk Protein vs Placebo 41

**Section 11 – PRISMA NMA Checklist of Items to Include When Reporting A Systematic Review Involving a Network Meta-analysis 42**

**Section 11 – Terminology**

Box 1. Terminology: Reviews With Networks of Multiple Treatments

Network meta-analysis (NMA), also referred to as multiple-treatments meta-analysis, is an extension of conventional pairwise meta-analysis that allows the simultaneous comparison of more than two interventions within a single analytical framework. By integrating direct evidence (from head-to-head trials) and indirect evidence (via a common comparator), NMA enables the estimation of relative treatment effects even when some interventions have not been directly compared.

A key assumption underlying NMA is transitivity, which requires that the included trials are sufficiently comparable in terms of clinical and methodological characteristics across treatment comparisons. Another fundamental assumption is consistency, defined as the statistical agreement between direct and indirect estimates for the same comparison within the network.

Treatment ranking can be summarized using probabilistic measures, such as the Surface Under the Cumulative Ranking curve (SUCRA), which reflects the probability that an intervention is among the most effective options for a given outcome. Together, these concepts support a comprehensive and coherent synthesis of evidence across complex intervention networks.

**Section 2 – Search Strategy**

2.1 Detailed Search Strategy

2.1.1 Table S1: Search Strategy in PubMed

| #1  ("Whey Proteins"[Mesh] OR Protein, Whey OR Proteins, Whey OR Whey Protein OR "Soybean Proteins"[Mesh] OR Soy Bean Proteins OR Soybean Protein OR Protein, Soybean OR Soy Bean Protein OR Bean Protein, Soy OR Protein, Soy Bean OR Dietary Soybean Proteins OR Dietary Soybean Protein OR Protein, Dietary Soybean OR Proteins, Dietary Soybean OR Soybean Protein, Dietary OR Soybean Proteins, Dietary OR Soy Proteins OR Protein, Soy OR Proteins, Soy OR Soy Protein OR "Meat Proteins"[Mesh] OR Meat Protein OR "Plant Proteins"[Mesh] OR Proteins, Plant OR Plant Protein OR Protein, Plant OR "Egg Proteins"[Mesh] OR Proteins, Egg OR Ovum Protein OR Protein, Ovum OR Ovum Proteins OR Proteins, Ovum OR Egg Protein OR Protein, Egg OR Egg White Proteins OR Proteins, Egg White OR White Proteins, Egg OR Egg White Protein OR Protein, Egg White OR White Protein, Egg OR Yolk Proteins OR Proteins, Yolk OR Egg Yolk Proteins OR Proteins, Egg Yolk OR Yolk Proteins, Egg OR Yolk Protein OR Protein, Yolk OR Egg Yolk Protein OR Protein, Egg Yolk OR Yolk Protein, Egg OR Egg Shell Proteins OR Proteins, Egg Shell OR Shell Proteins, Egg OR Egg Shell Protein OR Protein, Egg Shell OR Shell Protein, Egg OR "Collagen"[Mesh] OR Avicon OR Avitene OR Collagen Felt OR Zyderm OR Collastat OR Dermodress OR Microfibril Collagen Hemostat OR Collagen Hemostat, Microfibril OR Pangen OR alpha-Collagen OR alpha Collagen OR Collagen Fleece OR Collagenfleece OR ("Caseins"[Mesh] OR Casein OR alpha(S1)-Casein C OR Sodium Caseinate OR Caseinate, Sodium OR alpha(S2)-Casein OR epsilon-Casein OR epsilon Casein OR alpha-Caseins OR alpha Caseins OR alpha-Casein OR alpha Casein OR beta-Caseins OR beta Caseins OR beta-Casein OR beta Casein OR Casein A OR gamma-Caseins OR gamma Caseins OR gamma-Casein OR gamma Casein OR K-Casein OR K Casein OR kappa-Caseins OR kappa Caseins OR kappa-Casein OR kappa Casein OR alpha(S1)-Casein OR alpha(S1)-Casein A OR Acetylated, Dephosphorylated beta-Casein OR beta-Casein Acetylated, Dephosphorylated OR AD beta-Casein OR AD beta Casein OR beta-Casein, AD OR alpha(S1)-Casein B) |
| --- |
| #2  "Exercise"[Mesh] OR Exercises OR Physical Activity OR Activities, Physical OR Activity, Physical OR Physical Activities OR Exercise, Physical OR Exercises, Physical OR Physical Exercise OR Physical Exercises OR Acute Exercise OR Acute Exercises OR Exercise, Acute OR Exercises, Acute OR Exercise, Isometric OR Exercises, Isometric OR Isometric Exercises OR Isometric Exercise OR Exercise, Aerobic OR Aerobic Exercise OR Aerobic Exercises OR Exercises, Aerobic OR Exercise Training OR Exercise Trainings OR Training, Exercise OR Trainings, Exercise OR "Muscle Strength"[Mesh] OR Strength, Muscle OR Arthrogenic Muscle Inhibition OR Arthrogenic Muscle Inhibitions OR Inhibition, Arthrogenic Muscle OR Muscle Inhibition, Arthrogenic OR "Recovery of Function"[Mesh] OR Function Recoveries OR Function Recovery OR "Resistance Training"[Mesh] OR Training, Resistance OR Strength Training OR Training, Strength OR Weight-Lifting Strengthening Program OR Strengthening Program, Weight-Lifting OR Strengthening Programs, Weight-Lifting OR Weight Lifting Strengthening Program OR Weight-Lifting Strengthening Programs OR Weight-Lifting Exercise Program OR Exercise Program, Weight-Lifting OR Exercise Programs, Weight-Lifting OR Weight Lifting Exercise Program OR Weight-Lifting Exercise Programs OR Weight-Bearing Strengthening Program OR Strengthening Program, Weight-Bearing OR Strengthening Programs, Weight-Bearing OR Weight Bearing Strengthening Program OR Weight-Bearing Strengthening Programs OR Weight-Bearing Exercise Program OR Exercise Program, Weight-Bearing OR Exercise Programs, Weight-Bearing OR Weight Bearing Exercise Program OR Weight-Bearing Exercise Programs OR "Body Composition"[Mesh] OR Body Compositions OR Composition, Body OR Compositions, Body OR "Hypertrophy"[Mesh] OR Hypertrophies) |
| randomizedcontrolledtrial[Filter] |
| #1 AND #2 |

2.1.2 Table S2: Search Strategy in Scopus

| #1  ( TITLE-ABS-KEY ( "whey protein" OR "beneprotein" OR "milk whey protein" OR "resource whey protein" OR "vitapro" OR "whey proteins" OR "soybean protein" OR "fujipuro" OR "soy bean protein" OR "soy protein" OR "soy proteins" OR "soya protein" OR "soybean proteins" OR "meat protein" OR "meat proteins" OR "plant protein" OR "fruit protein" OR "fruit proteins" OR "grain protein" OR "grain proteins" OR "leaf protein" OR "nut protein" OR "nut proteins" OR "plant proteins" OR "protein, vegetable" OR "vegetable protein" OR "vegetable proteins" OR "egg protein" OR "collagen" OR "biocor" OR "collagel" OR "collagen horm" OR "collastypt" OR "collistat" OR "lyostypt" OR "medistat" OR "novacol" OR "phonogel" OR "casein" OR "casein acid" OR "casein native" OR "caseine" OR "caseinogen" OR "deaminized casein" OR "phosphocasein" ) AND TITLE-ABS-KEY ( "exercise" OR "biometric exercise" OR "effort" OR "exercise capacity" OR "exercise performance" OR "exercise training" OR "exertion" OR "fitness training" OR "fitness workout" OR "physical conditioning, human" OR "physical effort" OR "physical exercise" OR "physical exertion" OR "physical work-out" OR "physical workout" OR "muscle strength" OR "dynamic strength, muscle" OR "muscle force" OR "muscle power" OR "muscular strength" OR "resistance training" OR "resistance exercise" OR "strength training" OR "body composition" OR "hypertrophy" ) AND TITLE-ABS-KEY ( randomized AND controlled AND trial ) ) AND ( LIMIT-TO ( EXACTKEYWORD , "randomized controlled trial" ) ) |
| --- |
| #2  TITLE-ABS-KEY ( "exercise" OR "biometric exercise" OR "effort" OR "exercise capacity" OR "exercise performance" OR "exercise training" OR "exertion" OR "fitness training" OR "fitness workout" OR "physical conditioning, human" OR "physical effort" OR "physical exercise" OR "physical exertion" OR "physical work-out" OR "physical workout" OR "muscle strength" OR "dynamic strength, muscle" OR "muscle force" OR "muscle power" OR "muscular strength" OR "resistance training" OR "resistance exercise" OR "strength training" OR "body composition" OR "hypertrophy" ) AND TITLE-ABS-KEY ( randomized AND controlled AND trial ) ) AND ( LIMIT-TO ( EXACTKEYWORD , "randomized controlled trial" ) ) |
| #1 AND #2 |

2.1.3 Table S3: Search Strategy in Embase

| #1  'whey protein'/exp OR 'beneprotein' OR 'milk whey protein' OR 'resource whey protein' OR 'vitapro' OR 'whey proteins' OR 'whey protein' OR 'soybean protein'/exp OR 'fujipuro' OR 'soy bean protein' OR 'soy protein' OR 'soy proteins' OR 'soya protein' OR 'soybean proteins' OR 'soybean protein' OR 'meat protein'/exp OR 'meat proteins' OR 'meat protein' OR 'plant protein'/exp OR 'fruit protein' OR 'fruit proteins' OR 'grain protein' OR 'grain proteins' OR 'leaf protein' OR 'nut protein' OR 'nut proteins' OR 'plant proteins' OR 'protein, vegetable' OR 'vegetable protein' OR 'vegetable proteins' OR 'plant protein' OR 'egg protein'/exp OR 'egg proteins' OR 'egg protein' OR 'collagen'/exp OR 'biocor' OR 'collagel' OR 'collagen horm' OR 'collastypt' OR 'collistat' OR 'lyostypt' OR 'medistat' OR 'novacol' OR 'phonogel' OR 'collagen' OR 'casein'/exp OR 'casein acid' OR 'casein native' OR 'caseine' OR 'caseinogen' OR 'caseins' OR 'deaminized casein' OR 'phosphocasein' OR 'casein' |
| --- |
| #2  'exercise'/exp OR 'biometric exercise' OR 'effort' OR 'exercise capacity' OR 'exercise performance' OR 'exercise training' OR 'exertion' OR 'fitness training' OR 'fitness workout' OR 'physical conditioning, human' OR 'physical effort' OR 'physical exercise' OR 'physical exertion' OR 'physical work-out' OR 'physical workout' OR 'exercise' OR 'muscle strength'/exp OR 'dynamic strength, muscle' OR 'dynamic strength, muscular' OR 'force, muscle' OR 'muscle dynamic strength' OR 'muscle force' OR 'muscle force velocity relationship' OR 'muscle power' OR 'muscular dynamic strength' OR 'muscular force' OR 'muscular power' OR 'muscular strength' OR 'strength, muscle' OR 'muscle strength' OR 'resistance training'/exp OR 'resistance exercise' OR 'resistance exercise training' OR 'resistance-type exercise' OR 'resistance-type training' OR 'strength training' OR 'strength-type exercise' OR 'strength-type training' OR 'resistance training' OR 'body composition'/exp OR 'composition, body' OR 'body composition' OR 'hypertrophy'/exp OR 'acral hypertrophy' OR 'hypertrophia' OR 'hypertrophy' |
| 3#  [embase]/lim NOT ([embase]/lim AND [medline]/lim) |
| #1 AND #2 AND #3 |

**Section 3 – Study Characteristics**

3.1 Table S4: Characteristics of the Included Studies – Intervention, Sample Size, and Age

| **Author, year** | **Intervention 1** | **Sample size** | **Mean age ± SD** | **Intervention 2** | **Sample size** | **Mean age ± SD** | **Intervention 3** | **Sample size** | **Mean age ± SD** | **Total mean age ± SD** |
| --- | --- | --- | --- | --- | --- | --- | --- | --- | --- | --- |
| Antonio et al., 2001 | Whey Protein | 11 | 21.5 ± 1.9 | Bovine Colostrum | 9 | 22.4 ± 1.4 |  |  |  |  |
| Arazi et al., 2011 | Whey Protein | 20 | 21.3 ± 1.2 | Placebo | 20 | 22.5 ± 3.4 |  |  |  |  |
| Aristizabal et al., 2014 | Whey Protein | 18 | 22.8 ± 3.7 | Placebo | 22 | 22.3 ± 3.1 | Soy Protein | 21 | 24 ± 2.9 | 23 ± 3.3 |
| Babault et al., 2014 | Milk Protein | 22 | 22.5 ± 4.1 | Casein | 22 | 22.2 ± 3.9 | Placebo | 24 | 22 ± 3.9 |  |
| Babault et al., 2015 | Pea Protein | 47 | 22 ± 3.5 | Whey Protein | 46 | 22.1 ± 3.6 | Placebo | 44 | 21.7 ± 3.9 |  |
| Bach et al., 2022 | Whey Protein | 15 | 66.9 ± 4.3 | Placebo | 16 | 65.8 ± 5.0 |  |  |  |  |
| Balshaw et al., 2022 | Collagen | 19 | 27.0 ± 5.0 | Placebo | 20 | 24.4 ± 3.2 |  |  |  |  |
| Banaszek et al., 2019 | Whey Protein | 7 |  | Pea Protein | 8 |  |  |  |  | 38.7 ± 11.3 |
| Bemben et al., 2010 | Whey Protein | 11 | 58.2 ± 6.6 | Placebo | 10 | 56.1 ± 4.4 |  |  |  |  |
| Berger et al., 2014 | Soy Protein | 62 | 18.3 ± 0.4 | Casein | 58 | 18.2 ± 0.4 |  |  |  |  |
| Bijeh et al., 2022 | Soy Protein | 15 |  | Placebo | 15 |  |  |  |  | 65.6 ± 3.2 |
| Brinkworth et al., 2003 | Whey Protein | 17 | 23.8 ± 5.5 | Bovine Colostrum | 17 | 21.4 ± 3.4 |  |  |  |  |
| Brown et al., 2004 | Whey Protein | 9 | 20.3 ± 1.0 | Soy Protein | 9 | 21.6 ± 0.7 | Control | 9 | 20.4 ± 1.8 |  |
| Buckley et al., 2003 | Whey Protein | 25 | 25.1 ± 5.1 | Bovine Colostrum | 26 | 23.5 ± 4.1 |  |  |  |  |
| Burke et al., 2001 | Whey Protein | 10 |  | Placebo | 5 |  |  |  |  | 18–31 |
| Candow et al., 2006 | Whey Protein | 9 | 24.0 ± 6 | Placebo | 9 | 22.5 ± 6 | Soy Protein | 9 |  |  |
| Cooke et al., 2010 | Whey Protein | 9 | 24 ± 5 | Placebo | 8 | 22 ± 4 |  |  |  |  |
| Cribb et al., 2006A | Whey Protein | 6 | 27 ± 7 | Casein | 7 | 26 ± 5 |  |  |  |  |
| Cribb et al., 2006B | Whey Protein | 5 | 24 ± 5 | Placebo | 7 | 24 ± 7 |  |  |  |  |
| Davies et al., 2020 | Whey Protein | 7 | 24 ± 4 | Control | 8 | 23 ± 5 |  |  |  |  |
| Deibert et al., 2011 | Soy Protein | 13 | 55.9 ± 3.5 | Placebo | 13 | 55.5 ± 4.8 |  |  |  |  |
| Dirks et al., 2017 | Milk Protein | 17 | 77 ± 8.2 | Placebo | 17 | 76 ± 8.2 |  |  |  |  |
| Duarte et al., 2019 | Whey Protein | 4 | 25 ± 5.2 | Placebo | 4 | 21 ± 2.7 |  |  |  |  |
| Duff et al., 2014 | Whey Protein | 13 | 57.5 ± 6.3 | Bovine Colostrum | 12 | 61.8 ± 4.8 |  |  |  |  |
| Dulac et al., 2021 | Whey Protein | 21 |  | Placebo | 19 |  | Casein | 20 |  | 69 ± 7 |
| Eliot et al., 2008 | Whey Protein | 11 |  | Placebo | 10 |  |  |  |  | 48–72 |
| Erskine et al., 2012 | Whey Protein | 17 | 23.1 ± 3.0 | Placebo | 16 | 23.7 ± 2.9 |  |  |  |  |
| Farnfield et al., 2012 | Whey Protein | 17 | 45.7 ± 24.7 | Placebo | 17 | 45.2 ± 24.3 |  |  |  |  |
| Fernandes et al., 2018 | Whey Protein | 16 | 67 ± 4.1 | Placebo | 16 | 67.8 ± 4.0 |  |  |  |  |
| Griffen et al., 2021 | Whey Protein | 9 | 67 ± 3 | Placebo | 8 | 67 ± 2.82 |  |  |  |  |
| Griffen et al., 2022 | Whey Protein | 9 | 68 ± 3 | Placebo | 9 | 67 ± 3 |  |  |  |  |
| Hamarsland et al., 2018 | Whey Protein | 18 |  | Control | 18 |  |  |  |  | 29 ± 6 |
| Hamarsland et al., 2019 | Whey Protein | 15 | 74 ± 3.6 | Control | 15 | 72.9 ± 1.8 |  |  |  |  |
| Hartman et al., 2007 | Soy Protein | 19 |  | Placebo | 19 |  | Milk Protein | 18 |  | 18–30 |
| Herda et al., 2013 | Whey Protein | 22 | 21 ± 1.6 | Placebo | 21 | 20.9 ± 1.7 | Control | 21 | 21.1 ± 2.4 |  |
| Herda et al., 2021 | Whey Protein | 46 | 62.2 ± 6.5 | Placebo | 55 | 61.7 ± 6.3 |  |  |  |  |
| Haun et al., 2018 | Whey Protein | 17 | 20.8 ± 1.6 | Soy Protein | 15 | 20.8 ± 1.7 | Placebo | 15 | 20.8 ± 1.1 |  |
| Hulmi et al., 2008 | Whey Protein | 13 | 31.4 ± 6.5 | Placebo | 14 | 36.4 ± 19.2 |  |  |  |  |
| Hulmi et al., 2015 | Whey Protein | 22 | 31.4 ± 6.5 | Placebo | 21 | 36.4 ± 19.2 |  |  |  |  |
| Joy et al., 2013 | Whey Protein | 12 |  | Rice Protein | 12 |  |  |  |  | 21.3 ± 1.9 |
| Junior et al., 2017 | Whey Protein | 15 | 67.4 ± 4.1 | Placebo | 16 | 67.8 ± 4.1 |  |  |  |  |
| Kim et al., 2023 | Whey Protein | 17 | 23,5 ± 2,7 | Placebo | 15 | 24,5 ± 3,3 |  |  |  | 24,0 ± 3,0 |
| Kirmse et al., 2019 | Collagen | 29 | 24 ± 2 | Placebo | 28 | 24 ± 3 |  |  |  |  |
| Kuwaba et al., 2023 | Collagen | 10 | 51.9 ± 5 | Placebo | 8 | 53.6 ± 7.3 |  |  |  |  |
| Lamb et al., 2020 | Peanut Protein | 20 | 60 ± 9 | Control | 19 | 58 ± 7 |  |  |  |  |
| Lockwood et al., 2016 | Whey Protein | 26 | 21.4 ± 2.8 | Placebo | 15 | 20.9 ± 1.5 |  |  |  |  |
| MackayPhillips et al., 2021 | Lactoalbumin | 15 |  | Placebo | 15 |  | Control | 15 |  | 30 ± 7 |
| McAdam et al., 2018 | Whey Protein | 34 | 19 ± 1 | Placebo | 35 | 19 ± 1 |  |  |  |  |
| Mobley et al., 2017 | Whey Protein | 17 | 21 ± 4.1 | Placebo | 15 | 21 ± 3.8 | Soy Protein | 15 | 21 ± 3.8 |  |
| Moon et al., 2020 | Whey Protein | 12 |  | Rice Protein | 12 |  |  |  |  | 32.8 ± 6.7 |
| Mori et al., 2018 | Whey Protein | 25 | 70.6 ± 4.2 | Control | 25 | 70.6 ± 4.2 |  |  |  |  |
| Nabuco et al., 2018 | Whey Protein | 21 | 66.2 ± 9.4 | Placebo | 23 | 66.5 ± 7.2 |  |  |  |  |
| Nabuco et al., 2019A | Whey Protein | 21 | 66.2 ± 9.4 | Placebo | 23 | 66.5 ± 7.1 |  |  |  |  |
| Nabuco et al., 2019B | Whey Protein | 21 | 66.2 ± 9.4 | Placebo | 23 | 66.5 ± 7.1 |  |  |  |  |
| Naclerio et al., 2017A | Whey Protein | 9 | 27.6 ± 5.2 | Placebo | 9 | 24.4 ± 7.1 | Beef | 9 | 25.6 ± 5.3 |  |
| Naclerio et al., 2017B | Whey Protein | 8 | 26 ± 5 | Placebo | 8 | 29 ± 9 | Beef | 8 | 25 ± 8 |  |
| Nakayama et al., 2020 | Milk Protein | 61 | 71.4 ± 6.2 | Placebo | 61 | 70.4 ± 5.4 |  |  |  |  |
| Obradovic et al., 2020 | Whey Protein | 10 | 23 ± 4 | Placebo | 10 | 23 ± 4 |  |  |  |  |
| Oertzen-Hagemann et al., 2019 | Collagen | 12 | 24.4 ± 2.3 | Placebo | 13 | 23.9 ± 2.9 |  |  |  |  |
| Ozan et al., 2020 | Whey Protein | 10 | 21.2 ± 2.4 | Placebo | 10 | 20.1 ± 2.1 | Control | 10 | 20.0 ± 1.3 |  |
| Rankin et al., 2004 | Milk Protein | 10 | 20.5 ± 1.9 | Control | 9 | 21 ± 1.4 |  |  |  |  |
| Reidy et al., 2016 | Whey Protein | 18 | 25 ± 4.6 | Placebo | 18 | 25 ± 4.7 |  |  |  |  |
| Reidy et al., 2017 | Whey Protein | 15 | 24.6 ± 3.8 | Placebo | 17 | 25.2 ± 4.1 |  |  |  |  |
| Rindom et al., 2016 | Whey Protein | 12 | 24.6 ± 2.1 | Collagen | 12 | 24.6 ± 2.1 |  |  |  |  |
| Roberson et al., 2021 | Whey Protein | 15 | 21 ± 2 | Placebo | 12 | 21 ± 1 |  |  |  |  |
| Sexton et al., 2021 | Peanut Protein | 23 | 21.7 ± 2 | Control | 24 | 21 ± 1.96 |  |  |  |  |
| Sharp et al., 2017 | Whey Protein | 10 | 19 ± 2 | Placebo | 10 | 21 ± 2 | Beef | 10 | 22 ± 4 |  |
| Taylor et al., 2016 | Whey Protein | 8 | 20 ± 2 | Placebo | 6 | 21 ± 3 |  |  |  |  |
| Thomson et al., 2015 | Soy Protein | 26 | 61.7 ± 8.3 | Control | 23 | 61.3 ± 6.9 |  |  |  |  |
| Vangsoe et al., 2018 | Insect Protein | 9 |  | Placebo | 6 |  |  |  |  | 24.2 ± 2.6 |
| Verdijk et al., 2009 | Casein | 13 |  | Placebo | 13 |  |  |  |  | 72 ± 10.5 |
| Volek et al., 2013 | Whey Protein | 19 | 22.8 ± 3.7 | Placebo | 22 | 22.3 ± 3.1 | Soy Protein | 22 | 24 ± 2.9 |  |
| Watanabe et al., 2008 | Fish Protein | 12 | 69.8 ± 5.5 | Placebo | 13 | 68.3 ± 5.9 |  |  |  |  |
| Weisgarber et al., 2012 | Whey Protein | 9 | 24.5 ± 1.8 | Placebo | 8 | 23.4 ± 4.4 |  |  |  |  |
| West et al., 2017 | Whey Protein | 12 |  | Placebo | 12 |  |  |  |  | 24 ± 4 |
| Wilborn et al., 2013 | Whey Protein | 8 | 20 ± 1.9 | Casein | 8 | 21 ± 2.8 |  |  |  |  |
| Zbinden-Foncea et al., 2023 | Whey Protein | 6 |  | Placebo | 6 |  |  |  |  | 22.4 ± 3.1 |

Legend: SD = Standard deviation; N/a = Not assessed.

3.2 Table S5: Included Studies in the Review – Intervention, Dose/Day, and Follow-Up

| **Author, year** | **Intervention 1** | **Dose 1** | **Intervention 2** | **Dose 2** | **Intervention 3** | **Dose 3** | **Training Session/Week** | **Follow up** |
| --- | --- | --- | --- | --- | --- | --- | --- | --- |
| Antonio et al., 2001 | Whey Protein | 20 g | Bovine Colostrum | 20 g |  |  | 3x/week | 8 weeks |
| Arazi et al., 2011 | Whey Protein | 20 g | Placebo (Amido) | 20 g |  |  | 3 x | 8 weeks |
| Aristizabal et al., 2014 | Whey Protein | 21.6 g WP22.5 g maltodextrin | Placebo | 45.2 g maltodextrin | Soy Protein | 20 g soy 24.5 g maltodextrin | 3 x | 9 months |
| Babault et al., 2014 | Milk Protein | 30 g | Casein | 30g | Placebo | 30 g (10.5 g sucrose, 19.5 g maltodextri) | 3x | 10 weeks |
| Babault et al., 2015 | Pea Protein | 25 g | Whey Protein | 25g | Placebo | 25g maltodextrin | 3x | 12 weeks |
| Bach et al., 2022 | Whey Protein | 40 g | Placebo | 40 g maltodextrin |  |  | 2 x | 12 weeks |
| Balshaw et al., 2022 | Collagen | 15 g | Placebo | 15 g maltodextrin |  |  | 3 x | 15 weeks |
| Banaszek et al., 2019 | Whey Protein | 24 g | Pea Protein | 24 g |  |  | 4 x | 8 weeks |
| Bemben et al., 2010 | Whey Protein | 35 g | Placebo | 480 ml Gatorade |  |  | 3 x | 14 weeks |
| Berger et al., 2014 | Soy Protein | 20 g | Casein | 20 g |  |  | 3 x | 16 weeks |
| Bijeh et al., 2022 | Soy Protein | 240 ml soy milk | Placebo | 240 ml artificially sweetened-water |  |  | 3 x | 12 weeks |
| Brinkworth et al., 2003 | Bovine Colostrum | 60 g | Whey Protein | 60 g |  |  | 4 x | 8 weeks |
| Brown et al., 2004 | Whey Protein | 33 g | Soy Protein | 33 g | Control |  | 3 x | 9 weeks |
| Buckley et al., 2003 | Bovine Colostrum | 60 g | Whey Protein | 60 g |  |  | 3 x | 8 weeks |
| Burke et al., 2001 | Whey Protein | 1.2 g/kg/dia | Placebo | 1.2 g/kg/dia maltodextrin |  |  | 4 x | 12 weeks |
| Candow et al., 2006 | Whey Protein | 1.2 g/kg/dia | Placebo | 1.2 g/kg/dia maltodextrin | Soy Protein | 1.2 g/kg/dia | 3 x | 6 weeks |
| Cooke et al., 2010 | Whey Protein | 1.5 g/kg/dia | Placebo | 1.5 g/kg/dia dextrose |  |  | 1 session | 2 weeks |
| Cribb et al., 2006A | Whey Protein | 1.5 g/kg/dia | Casein | 1.5 g/kg/dia |  |  | 3 x | 10 weeks |
| Cribb et al., 2006B | Whey Protein | 1.5 g/kg/dia | Placebo | 1.5 g/kg/dia |  |  | 3-5 x | 11 weeks |
| Davies et al., 2020 | Whey Protein | 0.33 g/kg/dia | Control | 0.33 g/kg/dia (water and identically flavored to match the whey protein supplement) |  |  | 3x | 1 week |
| Deibert et al., 2011 | Soy Protein | 50 g | Placebo |  |  |  | 2x | 12 weeks |
| Dirks et al., 2017 | Milk Protein | 15 g | Placebo | 15 g lactose |  |  | 2x | 24 weeks |
| Duarte et al., 2019 | Whey Protein | 30 g | Placebo | 35 g amylopectin and cocoa |  |  | 3 x | 12 weeks |
| Duff et al., 2014 | Whey Protein | 60 g | Bovine Colostrum | 60 g |  |  | 3x | 8 weeks |
| Dulac et al., 2021 | Whey Protein | 30 g | Placebo | 19.4 g maltodextrin | Casein | 30 g | 3 x | 12 weeks |
| Eliot et al., 2008 | Whey Protein | 35 g | Placebo | 480 ml Gatorade |  |  | 3 x | 14 weeks |
| Erskine et al., 2012 | Whey Protein | 40 g | Placebo | 13.6 g lactose |  |  | 3 x | 12 weeks |
| Farnfield et al., 2012 | Whey Protein | 27 g | Placebo | 200 ml with the same artificial flavorings and sweetener |  |  | 3 x | 12 weeks |
| Fernandes et al., 2018 | Whey Protein | 35 g | Placebo | 35 g maltodextrin |  |  | 3x | 12 weeks |
| Griffen et al., 2021 | Whey Protein | 25 g | Placebo | 23.7 g maltodextrin |  |  | 2 x | 12 weeks |
| Griffen et al., 2022 | Whey Protein | 25 g | Placebo | 23.7 g maltodextrin |  |  | 2 x | 12 weeks |
| Hamarsland et al., 2018 | Whey Protein | 40 g | Control | Milk 38.6g protein |  |  | 3 x | 12 weeks |
| Hamarsland et al., 2019 | Whey Protein | 40 g | Control | Milk 39.4 g protein |  |  | 3 x | 11 weeks |
| Hartman et al., 2007 | Soy Protein | 500 ml fluid soy | Placebo | 500 ml 9% maltodextrin | Milk Protein | 35 g protein | 5 x | 12 weeks |
| Herda et al., 2013 | Whey Protein | 20 g | Placebo | 27 g maltodextrin |  |  | 3 x | 8 weeks |
| Herda et al., 2021 | Whey Protein | 20 g WP | Placebo | 15 g maltodextrin |  |  | 3 x | 12 weeks |
| Haun et al., 2018 | Whey Protein | 53 g | Soy Protein | 53 g | Placebo | 75 g maltodextrin | 3x | 12 weeks |
| Hulmi et al., 2008 | Whey Protein | 37.5 g | Placebo | 34.5 g maltodextrin |  |  | 2-3 x | 12 weeks |
| Hulmi et al., 2015 | Whey Protein | 30 g | Placebo | 30 g maltodextrin |  |  | 2-3 x | 12 weeks |
| Hwang et al., 2017 | Whey Protein | 25 g | Placebo | 25 g maltodextrin |  |  |  | 10 weeks |
| Joy et al., 2013 | Whey Protein | 48 | Rice Protein | 28 g |  |  | 3 x | 8 weeks |
| Junior et al., 2017 | Whey Protein | 35 g | Placebo | 35 g maltodextrin |  |  | 3 x | 12 weeks |
| Kim et al., 2023 | Whey Protein | 60 g | Placebo | 75 g maltodextrin |  |  | 6 x | 4 weeks |
| Kirmse et al., 2019 | Collagen | 15 g | Placebo | 15 g Sipernat 350 |  |  | 3 x | 12 weeks |
| Kuwaba et al., 2023 | Collagen | 10 g | Placebo | 10 g dextrin |  |  | 1 session | 13 weeks |
| Lamb et al., 2020 | Peanut Protein | 75 g | Control |  |  |  | 2x | 6 to 10 weeks |
| Lockwood et al., 2016 | Whey Protein | 60 g | Placebo | 60 g dextrose |  |  | 4x | 8 weeks |
| MackayPhillips et al., 2021 | Lactoalbumin | 40 g | Placebo | 40 g corn starch | Control | 40 g Zein | 3 sessions | 3 weeks |
| McAdam et al., 2018 | Whey Protein | 77 g | Placebo | 127 g dextrose |  |  |  | 8 weeks |
| Mobley et al., 2017 | Whey Protein | 53 g | Placebo | 88 g maltodextrin | Soy Protein | 79.4 g | 3 x | 12 weeks |
| Moon et al., 2020 | Whey Protein | 24 g | Rice Protein | 24 g |  |  |  | 8 weeks |
| Mori et al., 2018 | Whey Protein | 25 g | Control |  |  |  | 2x | 24 weeks |
| Nabuco et al., 2018 | Whey Protein | 35 g | Placebo | 35 g maltodextrin |  |  | 3 x | 8 weeks |
| Nabuco et al., 2019A | Whey Protein | 35 g | Placebo | 35 g maltodextrin |  |  | 3 x | 12 weeks |
| Nabuco et al., 2019B | Whey Protein | 35 g | Placebo | 35 g maltodextrin |  |  | 3 x | 12 weeks |
| Naclerio et al., 2017A | Whey Protein | 20 g WP | Placebo | 20 g maltodextrin | Beef | 20 g | 3 x | 10 weeks |
| Naclerio et al., 2017B | Whey Protein | 20 g WP | Placebo | 20 g maltodextrin | Beef | 20g | 3 x | 8 weeks |
| Nakayama et al., 2020 | Milk Protein | 10 g | Placebo | 10 g maltodextrin |  |  | 7 x | 26 weeks |
| Obradovic et al., 2020 | Whey Protein | 1 g/kg | Placebo | 10 g maltodextrin |  |  | 4 x | 8 weeks |
| Oertzen-Hagemann et al., 2019 | Collagen | 15 g | Placebo | 15 g silicon dioxide |  |  | 3 x | 12 weeks |
| Ozan et al., 2020 | Whey Protein | 1.5 g/kg | Placebo | 3 g/kg maltodextrin | Control |  | 3x | 10 weeks |
| Rankin et al., 2004 | Milk Protein | 0.21 g/kg | Control | 1.25 g/kg maltodextrin |  |  | 3x | 10 weeks |
| Reidy et al., 2016 | Whey Protein | 22 g | Placebo | 22g maltodextrin |  |  | 3 x | 12 weeks |
| Reidy et al., 2017 | Whey Protein | 22 g | Placebo | 22 g maltodextrin |  |  | 3 x | 12 weeks |
| Rindom et al., 2016 | Whey Protein | 25 g | Collagen | 25 g |  |  | 5 x | 7 weeks |
| Roberson et al., 2021 | Whey Protein | 26 g | Placebo | 44.4 g maltodextrin |  |  | 3 x | 12 weeks |
| Sexton et al., 2021 | Peanut Protein | 75 g | Control |  |  |  | 2 x | 10 weeks |
| Sharp et al., 2017 | Whey Protein | 46 g | Placebo | 46 g maltodextrin | Beef | 46 g | 4 x | 8 weeks |
| Taylor et al., 2016 | Whey Protein | 48 g | Placebo | 48 g maltodextrina |  |  | 4 x | 8 weeks |
| Thomson et al., 2015 | Soy Protein | 27 g | Control | 27 g protein (milk) |  |  | 3 x | 12 weeks |
| Vangsoe et al., 2018 | Insect Protein | 0.4 g/kg | Placebo | 0.4 g/kg maltodextrin |  |  | 4 x | 8 weeks |
| Verdijk et al., 2009 | Casein | 20 g | Placebo | Flavored water |  |  | 3 x | 12 weeks |
| Volek et al., 2013 | Whey Protein | 21.6 g | Placebo | 45.2 maltodextrin | Soy Protein | 20 g | 3 x | 36 weeks |
| Watanabe et al., 2008 | Fish Protein | 180 g | Placebo | 150 g dextrin |  |  | 2x | 6 weeks |
| Weisgarber et al., 2012 | Whey Protein | 0.3 g/kg | Placebo | 0.2 g/kg maltodextrin, 0.1 g/kg sucrose |  |  | 4 x | 8 weeks |
| West et al., 2017 | Whey Protein | 50 g WP | Placebo | 50 g maltodextrin |  |  |  | 24 h |
| Wilborn et al., 2013 | Whey Protein | 48 g | Casein | 48 g |  |  | 4x | 8 weeks |
| Zbinden-Foncea et al., 2023 | Whey Protein | 30 g | Placebo | 0 g cho (juice) |  |  | 3 x | 8 weeks |

Legend: WP = Whey protein; CP = Collagen protein; C = Casein; PRO = Protein; CHO = Carbohydrate; Fat = Fat; Fiber = Fiber; Maltodextrin = Maltodextrin; LAC = Lactoalbumin; Corn starch = Corn starch; N/a = Not assessed.

**Section 4 – Risk of Bias Assessment**

4.1 Figure S1: Traffic Plots – Risk of Bias in the Included Studies – Strength Outcome
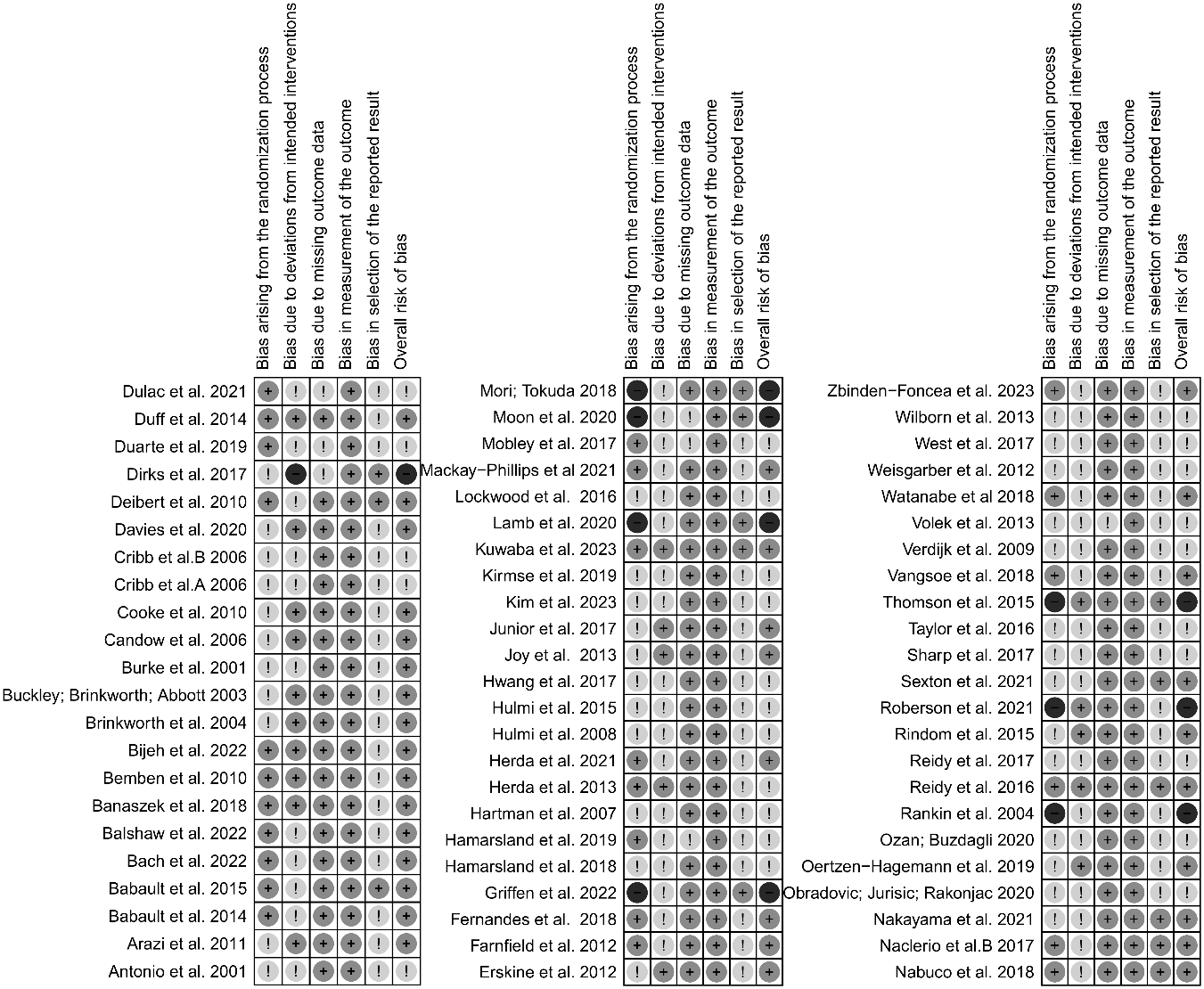


Legend: Visualization of the risk of bias assessment across the six domains of the Cochrane Risk of Bias 2.0 (RoB 2) tool for the strength outcome. Each row represents an included study, and each column corresponds to a specific bias domain:

(1) Bias arising from the randomization process;

(2) Bias due to deviations from intended interventions;

(3) Bias due to missing outcome data;

(4) Bias in measurement of the outcome;

(5) Bias in selection of the reported result;

(6) Overall risk of bias.

The color coding indicates:

Low risk of bias (dark gray); Some concerns (light gray); High risk of bias (black).

4.2 Figure S2: Traffic Plots – Risk of Bias in the Included Studies – Fat-Free Mass Outcome
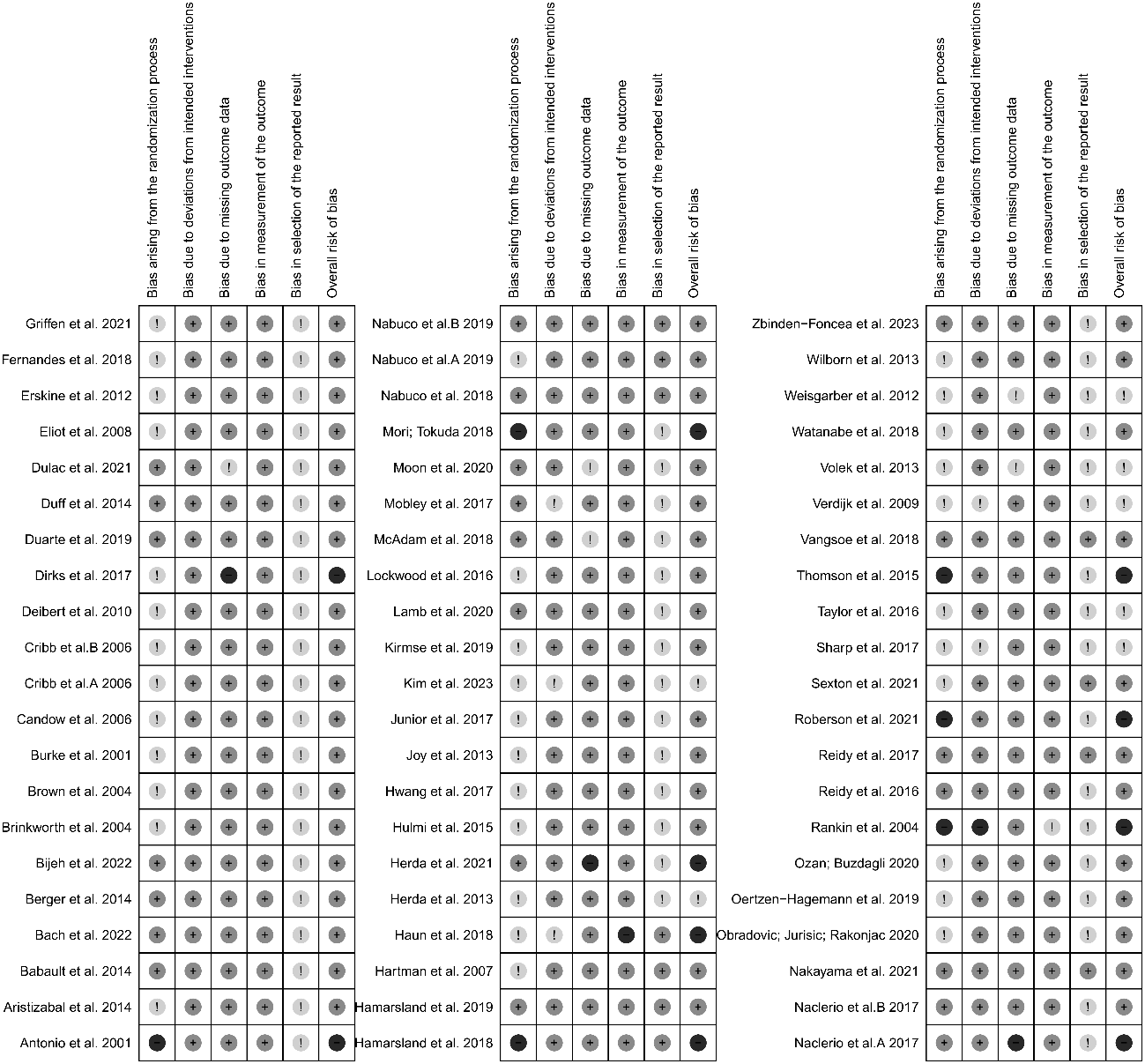


Legend: Visualization of the risk of bias assessment across the six domains of the Cochrane Risk of Bias 2.0 (RoB 2) tool for the strength outcome. Each row represents an included study, and each column corresponds to a specific bias domain:

(1) Bias arising from the randomization process;

(2) Bias due to deviations from intended interventions;

(3) Bias due to missing outcome data;

(4) Bias in measurement of the outcome;

(5) Bias in selection of the reported result;

(6) Overall risk of bias.

The color coding indicates:

Low risk of bias (dark gray); Some concerns (light gray); High risk of bias (black).

**Section 5 – Individual Study Results**

5.1 Table S6: Individual Results of the Included Studies

| **Author** | **Main_Findings** |
| --- | --- |
| Antonio et al., 2001 | The results showed that the group consuming bovine colostrum experienced a significant increase in bone-free lean mass (1.49 kg), while the placebo group showed a significant increase in body weight (2.11 kg). |
| Arazi et al., 2011 | The group supplemented with whey protein showed significant increases in body weight, muscle strength, explosive muscle power, and blood testosterone levels compared to the placebo group. |
| Aristizabal et al., 2014 | Nine months of resistance training significantly increased resting metabolic rate (~5% on average) in the whole cohort, with no significant differences between whey protein, soy protein, or carbohydrate placebo groups. |
| Babault et al., 2014 | Soluble milk protein supplementation (fast-digesting) significantly reduced resistance exercise-induced muscle fatigue compared with placebo and micellar casein. No significant between-group differences were observed in muscle thickness, strength, or endurance gains after 10 weeks of training. |
| Babault et al., 2015 | The results showed a significant increase in muscle thickness over time in all groups, with pea protein promoting a greater increase compared to the placebo. No significant differences were observed between pea protein and whey protein. |
| Bach et al., 2022 | Whey protein supplementation (20 g at breakfast and 20 g at dinner) in low-protein meals did not enhance resistance training–induced neuromuscular adaptations (i.e., strength and muscle mass) in healthy older adults. |
| Balshaw et al., 2022 | Collagen peptide supplementation produced a consistent set of effects indicating greater skeletal muscle remodeling after resistance training compared to the placebo. |
| Banaszek et al., 2019 | The intake of whey and pea proteins produced similar outcomes in physical adaptations after 8 weeks of high-intensity functional training. |
| Bemben et al., 2010 | Fourteen weeks of resistance training significantly increased muscular strength and lean body mass in middle-aged and older men, with no additional benefits from whey protein and/or creatine supplementation compared with placebo. |
| Berger et al., 2014 | Sixteen weeks of isoflavone-rich soy protein or casein-based meal replacement did not prevent significant weight gain in college-aged females; increases in body weight, fat mass, and fat-free soft tissue were similar between soy and casein groups. |
| Bijeh et al., 2022 | Twelve weeks of resistance training combined with daily soy milk consumption (240 mL) produced synergistic improvements in body composition (greater muscle mass gain and fat mass reduction), physical performance, and skeletal muscle regulatory markers (follistatin ↑, myostatin ↓, GDF11 ↓) in older men compared with resistance training or soy milk alone. |
| Brinkworth et al., 2003 | Bovine colostrum supplementation (60 g/day) during 8 weeks of unilateral elbow-flexor training produced significantly greater increases in arm circumference (2.3% vs. 0.0%) and total cross-sectional area (4.2% vs. -0.2%) of the trained arm compared to whey protein, mainly due to greater skin + subcutaneous fat area; no between-group differences were observed in strength gains. |
| Brown et al., 2004 | Both soy and whey protein bar supplementation (33 g protein/day, 9 weeks) combined with resistance training significantly increased lean body mass in male weight trainees, whereas the training-only control group did not; soy preserved two markers of antioxidant status that declined in the whey and training-only groups. |
| Buckley et al., 2003 | Eight weeks of bovine colostrum supplementation (60 g/day) during strength and speed training significantly increased peak anaerobic power, but had no significant effect on alactic anaerobic work capacity or 1-repetition maximum strength. |
| Burke et al., 2001 | Six weeks of resistance training combined with whey protein (1.2 g/kg/day) significantly increased lean tissue mass and knee-extension peak torque compared to placebo; adding creatine monohydrate (0.1 g/kg/day) to whey produced the greatest increases in lean tissue mass and bench-press strength, but not all strength measures (e.g., squat, knee flexion) differed from placebo. |
| Candow et al., 2006 | Six weeks of resistance training combined with either whey or soy protein supplementation (1.2 g/kg/day) significantly increased lean tissue mass and strength compared to an isocaloric carbohydrate placebo; no significant differences were observed between whey and soy protein. |
| Cooke et al., 2010 | Isolated whey protein supplementation attenuated the reduction in isometric and isokinetic muscle strength during recovery compared to the carbohydrate control group. |
| Cribb et al., 2006A | Whey isolate supplementation resulted in significantly greater gains in lean body mass and strength, along with a significant reduction in fat mass, compared to casein. |
| Cribb et al., 2006B | Eleven weeks of resistance training combined with whey protein and/or creatine monohydrate (separately or together) produced significantly greater increases in strength, lean body mass, type I, IIa, and IIx fiber cross-sectional area, and contractile protein content compared to carbohydrate placebo; the combination of whey + creatine generally elicited the largest improvements. |
| Davies et al., 2020 | The results showed that whey protein supplementation (0.33 g/kg/day) did not increase the myofibrillar protein synthesis rate nor improve any parameter of muscle recovery (isometric squat strength, countermovement jump height, muscle soreness, and serum creatine kinase) throughout the training protocol days. |
| Deibert et al., 2011 | Twenty-four weeks of resistance training combined with soy protein supplementation (daily intake not specified in abstract) significantly improved body composition, strength, and several metabolic/hormonal parameters in previously untrained middle-aged men compared to resistance training alone or lifestyle counselling only. |
| Dirks et al., 2017 | Twenty-four weeks of resistance training with twice-daily milk protein supplementation (2 × 15 g) in frail elderly significantly increased type I (+23%) and type II (+34%) muscle fiber cross-sectional area compared to placebo; no changes occurred in satellite cell or myonuclear content in either group. |
| Duarte et al., 2019 | Isolated whey protein supplementation combined with resistance training may increase muscle mass without affecting muscle strength. Whey protein supplementation can alter body composition in favor of additional lean mass without significant changes in body fat. |
| Duff et al., 2014 | Eight weeks of resistance training combined with bovine colostrum supplementation (60 g/day) significantly increased leg press strength and reduced urinary N-telopeptides (a marker of bone resorption) compared to whey protein; both colostrum and whey protein groups similarly improved bench press strength, muscle thickness, lean tissue mass, bone mineral content, and cognitive function, with no changes in plasma IGF-1 or C-reactive protein. |
| Dulac et al., 2021 | Twelve weeks of mixed power training significantly improved lean mass, lower limb muscle strength and quality, functional capacity, muscle characteristics, and serum parameters in all groups (placebo, fast-digested whey protein, and slow-digested casein protein); adding 30 g/day of either fast- or slow-digested protein supplementation did not provide additional benefits beyond training alone in older men with adequate habitual protein intake. |
| Eliot et al., 2008 | Fourteen weeks of resistance training significantly improved regional arm fat (decrease), regional arm bone-free fat-free mass (increase), total body bone-free fat-free mass (increase), intracellular water (increase), and extracellular water (increase) in middle-aged men; supplementation with whey protein, creatine, or their combination had no additional effects on body composition changes compared to placebo. |
| Erskine et al., 2012 | Whey protein supplementation did not provide additional advantages over resistance training–induced gains in muscle strength and size. |
| Farnfield et al., 2012 | In the untrained state, resistance exercise combined with whey protein ingestion significantly increased phosphorylation of proteins involved in mRNA translation compared to exercise alone in both young and older men; after 12 weeks of training, this whey protein-induced phosphorylation response was reduced in older men but maintained in younger men. |
| Fernandes et al., 2018 | Twelve weeks of resistance training significantly improved HDL, LDL, triglycerides, total cholesterol, LDL/HDL ratio, and C-reactive protein in both groups; whey protein supplementation (35 g post-training) produced greater increases in lean soft tissue and training volume, and a greater reduction in total cholesterol/HDL ratio compared to placebo, with no group differences in waist circumference. |
| Griffen et al., 2021 | Twelve weeks of resistance exercise (twice weekly) significantly increased muscle strength, fat-free mass, and physical function, and decreased markers of systemic inflammation in healthy active older men; whey protein supplementation (50 g/day) alone significantly increased gait speed, with no synergistic effects observed when combining whey protein with resistance exercise |
| Griffen et al., 2022 | Twelve weeks of resistance exercise significantly increased fat-free mass, resting metabolic rate, sedentary energy expenditure, and sleeping metabolic rate compared to non-exercise in healthy older men; whey protein supplementation (50 g/day) significantly decreased fat mass and increased overnight protein oxidation, with no changes in total energy expenditure or respiratory exchange ratio. |
| Hamarsland et al., 2018 | Supplementation with milk powder or native whey protein during a 12-week strength training period did not result in significant differences in muscle mass and strength gains in young, untrained individuals. |
| Hamarsland et al., 2019 | Both groups, supplemented with native whey protein or milk, experienced significant increases in muscle mass and strength, with no significant differences between groups. |
| Hartman et al., 2007 | Twelve weeks of resistance training combined with immediate post-exercise consumption of fat-free milk significantly increased type II muscle fiber area, type I muscle fiber area (greater than control), and DXA-measured fat- and bone-free mass (greater than soy and control) compared to isoenergetic soy protein or carbohydrate; no between-group differences were observed in strength gains. |
| Herda et al., 2013 | Eight weeks of resistance training similarly increased muscle performance and size across all groups, regardless of protein supplementation. |
| Herda et al., 2021 | Twelve weeks of self-selected, self-paced resistance exercise plus 30 minutes of walking three times per week significantly improved resting heart rate, fat-free mass, percent body fat, handgrip strength, bench press strength, leg press strength, and all mobility measurements in both whey protein and placebo groups, with no differences between supplementation groups. |
| Huan et al., 2018 | Concentrated whey protein supplementation had the greatest effect on increasing the cross-sectional area of type II muscle fibers, while soy protein had the greatest effect on increasing the cross-sectional area of type I fibers. |
| Hulmi et al., 2008 | Twenty-one weeks of heavy resistance training combined with timed whey protein supplementation (15 g before and after each session) significantly increased vastus lateralis muscle cross-sectional area and cdk2 mRNA expression more than placebo; protein intake prevented 1-hour post-exercise decreases in myostatin and myogenin mRNA but did not affect activin receptor IIb, p21, FLRG, MAFbx, or MyoD expression. |
| Hulmi et al., 2015 | Post-exercise supplementation with whey protein, compared to carbohydrates or a combination of whey protein and carbohydrates, did not have a significant effect on increasing muscle mass or strength. However, the group that consumed whey protein experienced a greater reduction in total fat and abdominal area, resulting in a relatively greater increase in lean mass compared to the carbohydrate group. |
| Hwang et al., 2017 | After a resistance training program followed by a short detraining period and retraining, resistance-trained men maintained their acquired muscle strength regardless of whey protein supplementation. |
| Joy et al., 2013 | However, no significant differences were observed between the whey and rice protein groups regarding increases in lean body mass, muscle strength, or muscle thickness. |
| Junior et al., 2017 | Whey protein supplementation combined with resistance training in previously trained older women resulted in greater increases in muscle strength and hypertrophy compared to the placebo. |
| Kim et al., 2023 | Significant group interactions were observed in terms of increases in muscle mass, peak torque of the dominant knee flexors, dominant and non-dominant shoulder extensors, and total work of the dominant knee and shoulder extensors, with greater increases observed in the whey protein supplementation group. |
| Kuwaba et al., 2023 | Dietary collagen peptide intake may alleviate muscle soreness and fatigue, as well as improve muscle strength after exercise in middle-aged men, suggesting that collagen peptides are an effective and safe sports nutritional supplement. |
| Lamb et al., 2020 | Peanut protein powder supplementation, combined with a 6- to 10-week resistance training (RT) program, improves certain aspects of muscle hypertrophy and strength in older adults compared to the resistance training program alone.. |
| Lockwood et al., 2016 | Whey protein supplementation resulted in significant improvements in maximal strength and body composition compared to the placebo. |
| MackayPhillips et al., 2021 | Supplementation with 40 g of α-lactalbumin resulted in reduced handgrip strength stability and increased neuromuscular fatigue of the plantar flexors. |
| McAdam et al., 2018 | Whey protein supplementation during initial Army training resulted in significant improvements in push-up performance and reduced fat mass compared to the carbohydrate placebo group.. |
| Mobley et al., 2017 | After 12 weeks of resistance training, no significant differences were observed in muscle mass and strength gains among the groups supplemented with whey protein, soy protein, or leucine compared to the placebo. |
| Moon et al., 2020 | Daily intake of isonitrogenous doses of 24 g of rice protein or whey protein, combined with an eight-week resistance training program, led to similar changes in body composition and performance outcomes.. |
| Mori et al., 2018 | Whey protein supplementation after resistance exercise may be effective in preventing sarcopenia in healthy older women by improving muscle mass and physical function. |
| Nabuco et al., 2018 | Whey protein supplementation, whether taken before or after resistance training, promotes increases in skeletal muscle mass, muscle strength, and functional capacity in previously conditioned older women, regardless of the timing of supplementation relative to training. |
| Nabuco et al., 2019A | Whey protein supplementation (before or after training), combined with resistance training, promoted an increase in intracellular water and lean muscle tissue, along with a reduction in the extracellular-to-intracellular water ratio in previously conditioned older women. |
| Nabuco et al., 2019B | Whey protein supplementation, whether taken before or after resistance training, promoted an increase in appendicular lean soft tissue (ALST) and a reduction in the total cholesterol/HDL ratio in previously conditioned older women. |
| Naclerio et al., 2017A | Post-exercise supplementation with a protein- and carbohydrate-containing beverage (from beef or whey) produced resistance training and body composition outcomes similar to carbohydrate intake alone. |
| Naclerio et al., 2017B | The intake of carbohydrates alone or combined with beef or whey protein after resistance training results in similar training outcomes in terms of strength, body composition, and muscle hypertrophy in trained men. |
| Nakayama et al., 2020 | Milk protein supplementation after low- to moderate-intensity exercise may be effective in increasing muscle mass and improving body composition in healthy older adults. |
| Obradovic et al., 2020 | Whey protein supplementation during resistance training is more effective in increasing muscle strength compared to the placebo group. |
| Oertzen-Hagemann et al., 2019 | After 12 weeks of hypertrophy-focused resistance training combined with collagen peptide supplementation, participants who received the supplement showed a significant increase in lean body mass and muscle strength compared to the placebo group. |
| Ozan et al., 2020 | Both the whey protein and placebo groups showed an increase in the cross-sectional area of type I and type II muscle fibers after training. |
| Rankin et al., 2004 | In the male group, body fat mass increased in the control group but not in the peanut protein group. However, peak knee extensor torque increased in the control group but not in the peanut protein group. |
| Reidy et al., 2016 | Whey protein supplementation during 12 weeks of resistance training did not result in additional significant effects on lean mass, muscle strength, or muscle hypertrophy compared to the placebo. |
| Reidy et al., 2017 | Protein supplementation during resistance training has a modest effect on increasing lean body mass compared to training without supplementation. |
| Rindom et al., 2016 | Whey supplementation did not accelerate recovery compared with collagen.  Protein quality did not determine the restoration of strength, power, or endurance following intense resistance training. |
| Roberson et al., 2021 | Post-exercise consumption of milk and carbohydrate-electrolyte beverages resulted in similar adaptations to resistance training. |
| Sexton et al., 2021 | Post-exercise supplementation with high-quality proteins, whether from beef, chicken, or whey, significantly improves body composition and muscle strength in trained individuals. |
| Sharp et al., 2017 | Post-exercise consumption of beef, chicken, or whey protein leads to significant improvements in body composition by increasing lean mass and reducing body fat compared to the control group. |
| Taylor et al., 2016 | Whey protein supplementation, when combined with a resistance training program, was effective in increasing muscle mass, improving strength, and reducing fat mass in trained female athletes. |
| Thomson et al., 2015 | Soy protein supplementation attenuated muscle strength gains during resistance training in older adults compared to dairy protein intake or usual protein consumption. |
| Vangsoe et al., 2018 | Insect protein supplementation during eight weeks of resistance training promoted gains in muscle mass and strength in healthy young adults, with no significant differences in morphological adaptations such as hypertrophy or muscle strength between the supplementation groups. |
| Verdijk et al., 2009 | Timed protein supplementation before and after resistance exercise does not further augment skeletal muscle hypertrophy or strength gains in healthy elderly men consuming adequate dietary protein. |
| Volek et al., 2013 | Whey protein supplementation during resistance training significantly increases lean body mass compared to soy protein supplementation or isocaloric carbohydrate intake. |
| Watanabe et al., 2008 | The combination of resistance training and fish protein intake led to greater improvements in motor function, especially in motor units with a low recruitment threshold. There was also a change in the firing patterns of motor units with a high recruitment threshold. |
| Weisgarber et al., 2012 | Whey protein intake immediately before and during resistance exercise had no significant effect on muscle mass or strength in untrained young adults. |
| West et al., 2017 | Whey protein supplementation improves whole-body protein balance and performance recovery after resistance exercise. |
| Wilborn et al., 2013 | Pre- and post-exercise supplementation with whey and casein proteins, when combined with a periodized resistance training program, is capable of inducing significant changes in body composition and athletic performance in collegiate female athletes. No significant differences were observed between the effects of whey and casein proteins. |
| Zbinden-Foncea et al., 2023 | Neither chia flour supplementation nor whey protein supplementation resulted in additional improvements in body composition or strength gains following an 8-week resistance training program in healthy, untrained young adults. |

Legend: Summary of the main findings from the randomized controlled trials (RCTs) included in this network meta-analysis. The table presents the first author and year of publication, along with the key results reported by each study, focusing on the effects of protein supplementation combined with resistance training on muscle strength and lean body mass outcomes.

Abbreviations: RCTs = Randomized Controlled Trials; ALST = Appendicular Lean Soft Tissue; FFM = Fat-Free Mass; 1RM = One-Repetition Maximum.

**Section 6 – League Tables**

6.1 Table S7: League Table – Strength Outcome

| **Treatment** | **Beef** | **Bovine Colostrum** | **Casein** | **Collagen** | **Control** | **Fish protein** | **Insect protein** | **Lactoalbumin** | **Milk protein** | **Pea protein** | **Peanut protein** | **Placebo** | **Rice protein** | **Soy protein** | **Whey protein** |
| --- | --- | --- | --- | --- | --- | --- | --- | --- | --- | --- | --- | --- | --- | --- | --- |
| Beef | - | -0.05 [-0.7; 0.61] | 0.38 [-0.27; 1.02] | -0.18 [-0.82; 0.47] | 0.22 [-0.39; 0.83] | 0.23 [-0.74; 1.2] | 0.42 [-0.67; 1.51] | 0.04 [-0.8; 0.89] | 0.35 [-0.26; 0.97] | 0.06 [-0.6; 0.72] | 0.27 [-0.48; 1.01] | 0.23 [-0.34; 0.8] | 0.29 [-0.51; 1.1] | 0.14 [-0.48; 0.75] | 0.09 [-0.48; 0.66] |
| Bovine Colostrum | 0.05 [-0.61; 0.7] | - | 0.42 [-0.03; 0.88] | -0.13 [-0.59; 0.33] | 0.27 [-0.13; 0.67] | 0.28 [-0.58; 1.14] | 0.47 [-0.52; 1.46] | 0.09 [-0.62; 0.81] | 0.4 [-0.02; 0.82] | 0.11 [-0.36; 0.58] | 0.31 [-0.27; 0.9] | 0.28 [-0.07; 0.63] | 0.34 [-0.32; 1] | 0.19 [-0.23; 0.6] | 0.14 [-0.19; 0.46] |
| Casein | -0.38 [-1.02; 0.27] | -0.42 [-0.88; 0.03] | - | **-0.55 [-0.99; -0.11]** | -0.16 [-0.54; 0.22] | -0.14 [-0.98; 0.7] | 0.04 [-0.93; 1.02] | -0.33 [-1.03; 0.37] | -0.02 [-0.39; 0.34] | -0.31 [-0.77; 0.14] | -0.11 [-0.68; 0.46] | -0.14 [-0.45; 0.17] | -0.08 [-0.73; 0.57] | -0.24 [-0.63; 0.15] | -0.29 [-0.6; 0.03] |
| Collagen | 0.18 [-0.47; 0.82] | 0.13 [-0.33; 0.59] | **0.55 [0.11; 0.99]** | - | **0.39 [0.01; 0.78]** | 0.41 [-0.43; 1.25] | 0.6 [-0.38; 1.57] | 0.22 [-0.48; 0.92] | **0.53 [0.14; 0.92]** | 0.24 [-0.22; 0.69] | 0.44 [-0.13; 1.02] | **0.41 [0.1; 0.72]** | 0.47 [-0.19; 1.12] | 0.31 [-0.08; 0.71] | 0.26 [-0.06; 0.59] |
| Control | -0.22 [-0.83; 0.39] | -0.27 [-0.67; 0.13] | 0.16 [-0.22; 0.54] | **-0.39 [-0.78; -0.01]** | - | 0.02 [-0.8; 0.84] | 0.2 [-0.75; 1.16] | -0.17 [-0.81; 0.46] | 0.13 [-0.19; 0.45] | -0.16 [-0.56; 0.25] | 0.05 [-0.38; 0.47] | 0.01 [-0.22; 0.25] | 0.07 [-0.54; 0.69] | -0.08 [-0.38; 0.22] | -0.13 [-0.36; 0.1] |
| Fish protein | -0.23 [-1.2; 0.74] | -0.28 [-1.14; 0.58] | 0.14 [-0.7; 0.98] | -0.41 [-1.25; 0.43] | -0.02 [-0.84; 0.8] | - | 0.19 [-1.03; 1.4] | -0.19 [-1.2; 0.82] | 0.12 [-0.7; 0.94] | -0.17 [-1.03; 0.68] | 0.03 [-0.89; 0.96] | 0 [-0.79; 0.78] | 0.06 [-0.92; 1.03] | -0.1 [-0.92; 0.73] | -0.15 [-0.94; 0.65] |
| Insect protein | -0.42 [-1.51; 0.67] | -0.47 [-1.46; 0.52] | -0.04 [-1.02; 0.93] | -0.6 [-1.57; 0.38] | -0.2 [-1.16; 0.75] | -0.19 [-1.4; 1.03] | - | -0.38 [-1.5; 0.75] | -0.07 [-1.02; 0.89] | -0.36 [-1.35; 0.63] | -0.15 [-1.2; 0.89] | -0.19 [-1.11; 0.74] | -0.13 [-1.22; 0.97] | -0.28 [-1.24; 0.68] | -0.33 [-1.27; 0.6] |
| Lactoalbumin | -0.04 [-0.89; 0.8] | -0.09 [-0.81; 0.62] | 0.33 [-0.37; 1.03] | -0.22 [-0.92; 0.48] | 0.17 [-0.46; 0.81] | 0.19 [-0.82; 1.2] | 0.38 [-0.75; 1.5] | - | 0.31 [-0.36; 0.98] | 0.02 [-0.7; 0.73] | 0.22 [-0.54; 0.98] | 0.19 [-0.44; 0.82] | 0.25 [-0.6; 1.1] | 0.09 [-0.57; 0.76] | 0.04 [-0.59; 0.68] |
| Milk protein | -0.35 [-0.97; 0.26] | -0.4 [-0.82; 0.02] | 0.02 [-0.34; 0.39] | **-0.53 [-0.92; -0.14]** | -0.13 [-0.45; 0.19] | -0.12 [-0.94; 0.7] | 0.07 [-0.89; 1.02] | -0.31 [-0.98; 0.36] | - | -0.29 [-0.7; 0.12] | -0.09 [-0.62; 0.45] | -0.12 [-0.36; 0.12] | -0.06 [-0.68; 0.56] | -0.21 [-0.54; 0.11] | **-0.26 [-0.52; -0.01]** |
| Pea protein | -0.06 [-0.72; 0.6] | -0.11 [-0.58; 0.36] | 0.31 [-0.14; 0.77] | -0.24 [-0.69; 0.22] | 0.16 [-0.25; 0.56] | 0.17 [-0.68; 1.03] | 0.36 [-0.63; 1.35] | -0.02 [-0.73; 0.7] | 0.29 [-0.12; 0.7] | - | 0.21 [-0.38; 0.79] | 0.17 [-0.17; 0.51] | 0.23 [-0.43; 0.89] | 0.08 [-0.33; 0.49] | 0.03 [-0.31; 0.36] |
| Peanut protein | -0.27 [-1.01; 0.48] | -0.31 [-0.9; 0.27] | 0.11 [-0.46; 0.68] | -0.44 [-1.02; 0.13] | -0.05 [-0.47; 0.38] | -0.03 [-0.96; 0.89] | 0.15 [-0.89; 1.2] | -0.22 [-0.98; 0.54] | 0.09 [-0.45; 0.62] | -0.21 [-0.79; 0.38] | - | -0.04 [-0.52; 0.45] | 0.03 [-0.72; 0.77] | -0.13 [-0.65; 0.39] | -0.18 [-0.66; 0.3] |
| Placebo | -0.23 [-0.8; 0.34] | -0.28 [-0.63; 0.07] | 0.14 [-0.17; 0.45] | **-0.41 [-0.72; -0.1]** | -0.01 [-0.25; 0.22] | 0 [-0.78; 0.79] | 0.19 [-0.74; 1.11] | -0.19 [-0.82; 0.44] | 0.12 [-0.12; 0.36] | -0.17 [-0.51; 0.17] | 0.04 [-0.45; 0.52] | - | 0.06 [-0.52; 0.64] | -0.09 [-0.34; 0.15] | **-0.14 [-0.26; -0.03]** |
| Rice protein | -0.29 [-1.1; 0.51] | -0.34 [-1; 0.32] | 0.08 [-0.57; 0.73] | -0.47 [-1.12; 0.19] | -0.07 [-0.69; 0.54] | -0.06 [-1.03; 0.92] | 0.13 [-0.97; 1.22] | -0.25 [-1.1; 0.6] | 0.06 [-0.56; 0.68] | -0.23 [-0.89; 0.43] | -0.03 [-0.77; 0.72] | -0.06 [-0.64; 0.52] | - | -0.15 [-0.78; 0.47] | -0.2 [-0.77; 0.36] |
| Soy protein | -0.14 [-0.75; 0.48] | -0.19 [-0.6; 0.23] | 0.24 [-0.15; 0.63] | -0.31 [-0.71; 0.08] | 0.08 [-0.22; 0.38] | 0.1 [-0.73; 0.92] | 0.28 [-0.68; 1.24] | -0.09 [-0.76; 0.57] | 0.21 [-0.11; 0.54] | -0.08 [-0.49; 0.33] | 0.13 [-0.39; 0.65] | 0.09 [-0.15; 0.34] | 0.15 [-0.47; 0.78] | - | -0.05 [-0.3; 0.2] |
| Whey protein | -0.09 [-0.66; 0.48] | -0.14 [-0.46; 0.19] | 0.29 [-0.03; 0.6] | -0.26 [-0.59; 0.06] | 0.13 [-0.1; 0.36] | 0.15 [-0.65; 0.94] | 0.33 [-0.6; 1.27] | -0.04 [-0.68; 0.59] | **0.26 [0.01; 0.52]** | -0.03 [-0.36; 0.31] | 0.18 [-0.3; 0.66] | **0.14 [0.03; 0.26]** | 0.2 [-0.36; 0.77] | 0.05 [-0.2; 0.3] | - |

6.2 Table S8: League Table – Fat-Free Mass Outcome

| **Treatment** | **Beef** | **Bovine Colostrum** | **Casein** | **Collagen** | **Control** | **Fish protein** | **Insect protein** | **Milk protein** | **Peanut protein** | **Placebo** | **Rice protein** | **Soy protein** | **Whey protein** |
| --- | --- | --- | --- | --- | --- | --- | --- | --- | --- | --- | --- | --- | --- |
| Beef | - | 0.17 [-0.45; 0.79] | 0.34 [-0.19; 0.87] | -0.53 [-1.18; 0.13] | 0.35 [-0.18; 0.87] | 0.44 [-0.48; 1.35] | 0.4 [-0.64; 1.44] | 0.31 [-0.22; 0.83] | 0.39 [-0.29; 1.06] | 0.41 [-0.06; 0.89] | 0.36 [-0.38; 1.09] | 0.33 [-0.17; 0.83] | 0.25 [-0.22; 0.72] |
| Bovine Colostrum | -0.17 [-0.79; 0.45] | - | 0.17 [-0.31; 0.65] | **-0.7 [-1.32; -0.08]** | 0.17 [-0.3; 0.65] | 0.26 [-0.63; 1.15] | 0.23 [-0.79; 1.24] | 0.14 [-0.34; 0.62] | 0.21 [-0.42; 0.84] | 0.24 [-0.18; 0.66] | 0.19 [-0.51; 0.88] | 0.16 [-0.3; 0.61] | 0.08 [-0.33; 0.48] |
| Casein | -0.34 [-0.87; 0.19] | -0.17 [-0.65; 0.31] | - | **-0.87 [-1.39; -0.35]** | 0 [-0.33; 0.34] | 0.09 [-0.73; 0.92] | 0.06 [-0.9; 1.01] | -0.03 [-0.35; 0.29] | 0.04 [-0.5; 0.58] | 0.07 [-0.18; 0.32] | 0.02 [-0.61; 0.64] | -0.01 [-0.27; 0.24] | -0.09 [-0.35; 0.16] |
| Collagen | 0.53 [-0.13; 1.18] | **0.7 [0.08; 1.32]** | **0.87 [0.35; 1.39]** | - | **0.87 [0.35; 1.39]** | **0.96 [0.05; 1.87]** | 0.92 [-0.11; 1.96] | **0.83 [0.32; 1.35]** | **0.91 [0.24; 1.58]** | **0.94 [0.48; 1.4]** | **0.88 [0.15; 1.62]** | **0.85 [0.36; 1.35]** | **0.78 [0.31; 1.25]** |
| Control | -0.35 [-0.87; 0.18] | -0.17 [-0.65; 0.3] | 0 [-0.34; 0.33] | **-0.87 [-1.39; -0.35]** | - | 0.09 [-0.74; 0.91] | 0.05 [-0.91; 1.01] | -0.04 [-0.36; 0.29] | 0.04 [-0.39; 0.46] | 0.07 [-0.18; 0.32] | 0.01 [-0.6; 0.63] | -0.02 [-0.29; 0.26] | -0.1 [-0.34; 0.14] |
| Fish protein | -0.44 [-1.35; 0.48] | -0.26 [-1.15; 0.63] | -0.09 [-0.92; 0.73] | **-0.96 [-1.87; -0.05]** | -0.09 [-0.91; 0.74] | - | -0.04 [-1.25; 1.18] | -0.13 [-0.95; 0.69] | -0.05 [-0.98; 0.88] | -0.02 [-0.81; 0.76] | -0.08 [-1.05; 0.9] | -0.11 [-0.91; 0.7] | -0.18 [-0.98; 0.61] |
| Insect protein | -0.4 [-1.44; 0.64] | -0.23 [-1.24; 0.79] | -0.06 [-1.01; 0.9] | -0.92 [-1.96; 0.11] | -0.05 [-1.01; 0.91] | 0.04 [-1.18; 1.25] | - | -0.09 [-1.04; 0.86] | -0.01 [-1.06; 1.03] | 0.02 [-0.91; 0.94] | -0.04 [-1.13; 1.05] | -0.07 [-1.01; 0.87] | -0.15 [-1.08; 0.78] |
| Milk protein | -0.31 [-0.83; 0.22] | -0.14 [-0.62; 0.34] | 0.03 [-0.29; 0.35] | **-0.83 [-1.35; -0.32]** | 0.04 [-0.29; 0.36] | 0.13 [-0.69; 0.95] | 0.09 [-0.86; 1.04] | - | 0.08 [-0.46; 0.61] | 0.11 [-0.13; 0.34] | 0.05 [-0.57; 0.67] | 0.02 [-0.27; 0.31] | -0.06 [-0.31; 0.2] |
| Peanut protein | -0.39 [-1.06; 0.29] | -0.21 [-0.84; 0.42] | -0.04 [-0.58; 0.5] | **-0.91 [-1.58; -0.24]** | -0.04 [-0.46; 0.39] | 0.05 [-0.88; 0.98] | 0.01 [-1.03; 1.06] | -0.08 [-0.61; 0.46] | - | 0.03 [-0.46; 0.52] | -0.03 [-0.77; 0.72] | -0.06 [-0.56; 0.45] | -0.13 [-0.62; 0.35] |
| Placebo | -0.41 [-0.89; 0.06] | -0.24 [-0.66; 0.18] | -0.07 [-0.32; 0.18] | **-0.94 [-1.4; -0.48]** | -0.07 [-0.32; 0.18] | 0.02 [-0.76; 0.81] | -0.02 [-0.94; 0.91] | -0.11 [-0.34; 0.13] | -0.03 [-0.52; 0.46] | - | -0.06 [-0.63; 0.52] | -0.09 [-0.28; 0.11] | **-0.16 [-0.28; -0.05]** |
| Rice protein | -0.36 [-1.09; 0.38] | -0.19 [-0.88; 0.51] | -0.02 [-0.64; 0.61] | **-0.88 [-1.62; -0.15]** | -0.01 [-0.63; 0.6] | 0.08 [-0.9; 1.05] | 0.04 [-1.05; 1.13] | -0.05 [-0.67; 0.57] | 0.03 [-0.72; 0.77] | 0.06 [-0.52; 0.63] | - | -0.03 [-0.63; 0.57] | -0.11 [-0.67; 0.46] |
| Soy protein | -0.33 [-0.83; 0.17] | -0.16 [-0.61; 0.3] | 0.01 [-0.24; 0.27] | **-0.85 [-1.35; -0.36]** | 0.02 [-0.26; 0.29] | 0.11 [-0.7; 0.91] | 0.07 [-0.87; 1.01] | -0.02 [-0.31; 0.27] | 0.06 [-0.45; 0.56] | 0.09 [-0.11; 0.28] | 0.03 [-0.57; 0.63] | - | -0.08 [-0.28; 0.12] |
| Whey protein | -0.25 [-0.72; 0.22] | -0.08 [-0.48; 0.33] | 0.09 [-0.16; 0.35] | **-0.78 [-1.25; -0.31]** | 0.1 [-0.14; 0.34] | 0.18 [-0.61; 0.98] | 0.15 [-0.78; 1.08] | 0.06 [-0.2; 0.31] | 0.13 [-0.35; 0.62] | **0.16 [0.05; 0.28]** | 0.11 [-0.46; 0.67] | 0.08 [-0.12; 0.28] | - |

**Section 7 – Rankograms**

7.1 Figure S3: Rankogram – Strength Outcome


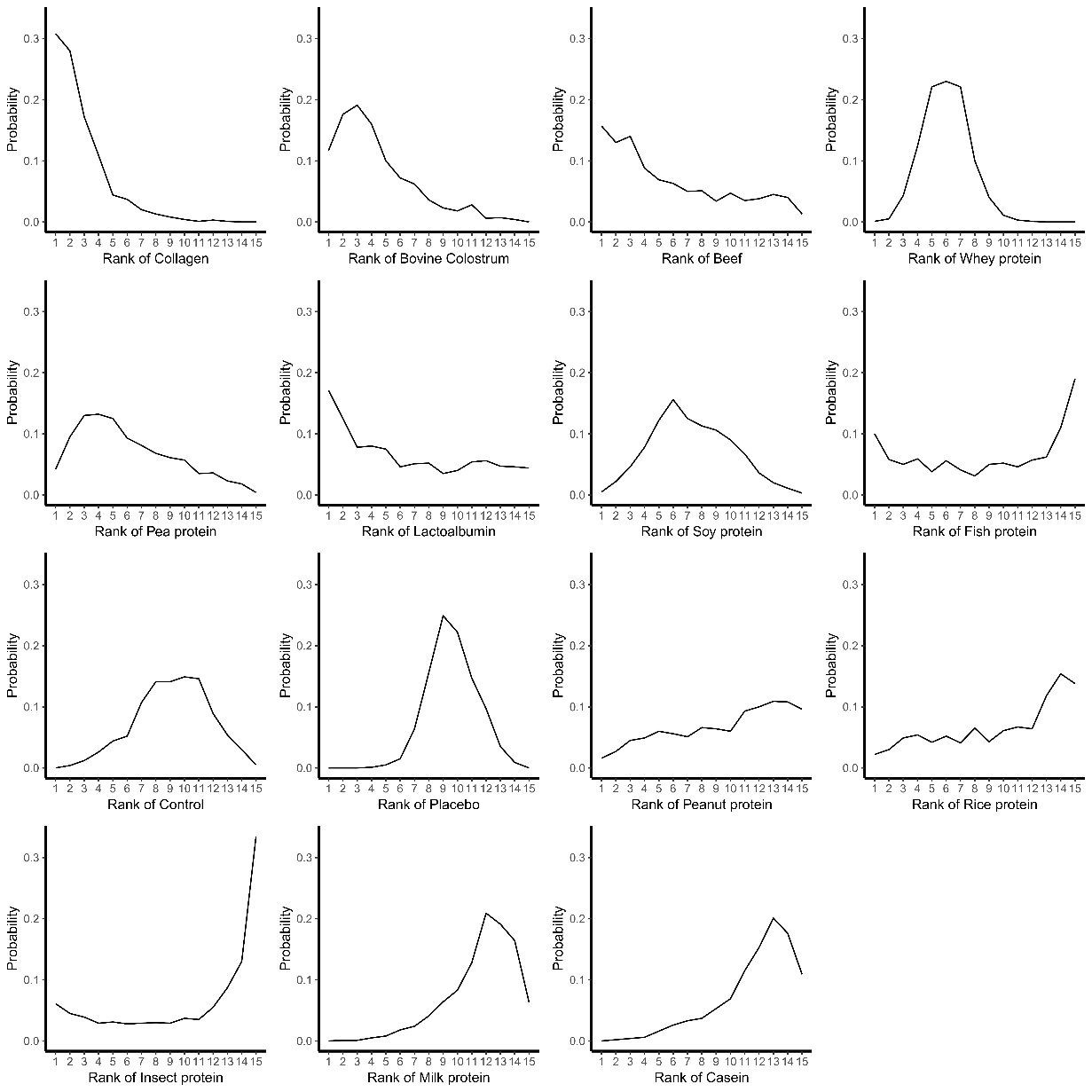


7.2 Figure S4: Rankogram – Fat-Free Mass Outcome


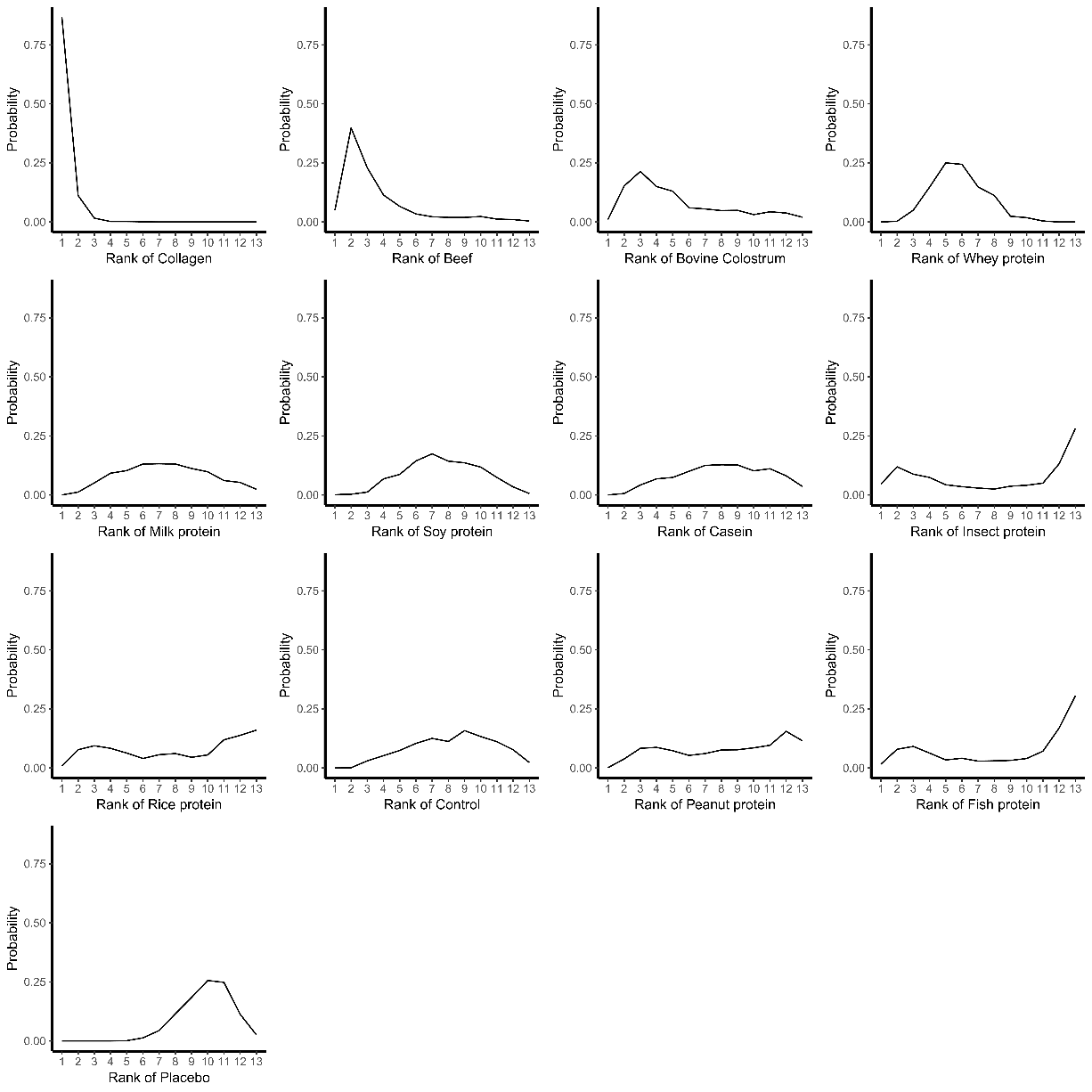


**Section 8 – CINeMA Assessment**

8.1 Figure S5: CINeMA for the Strength Outcome


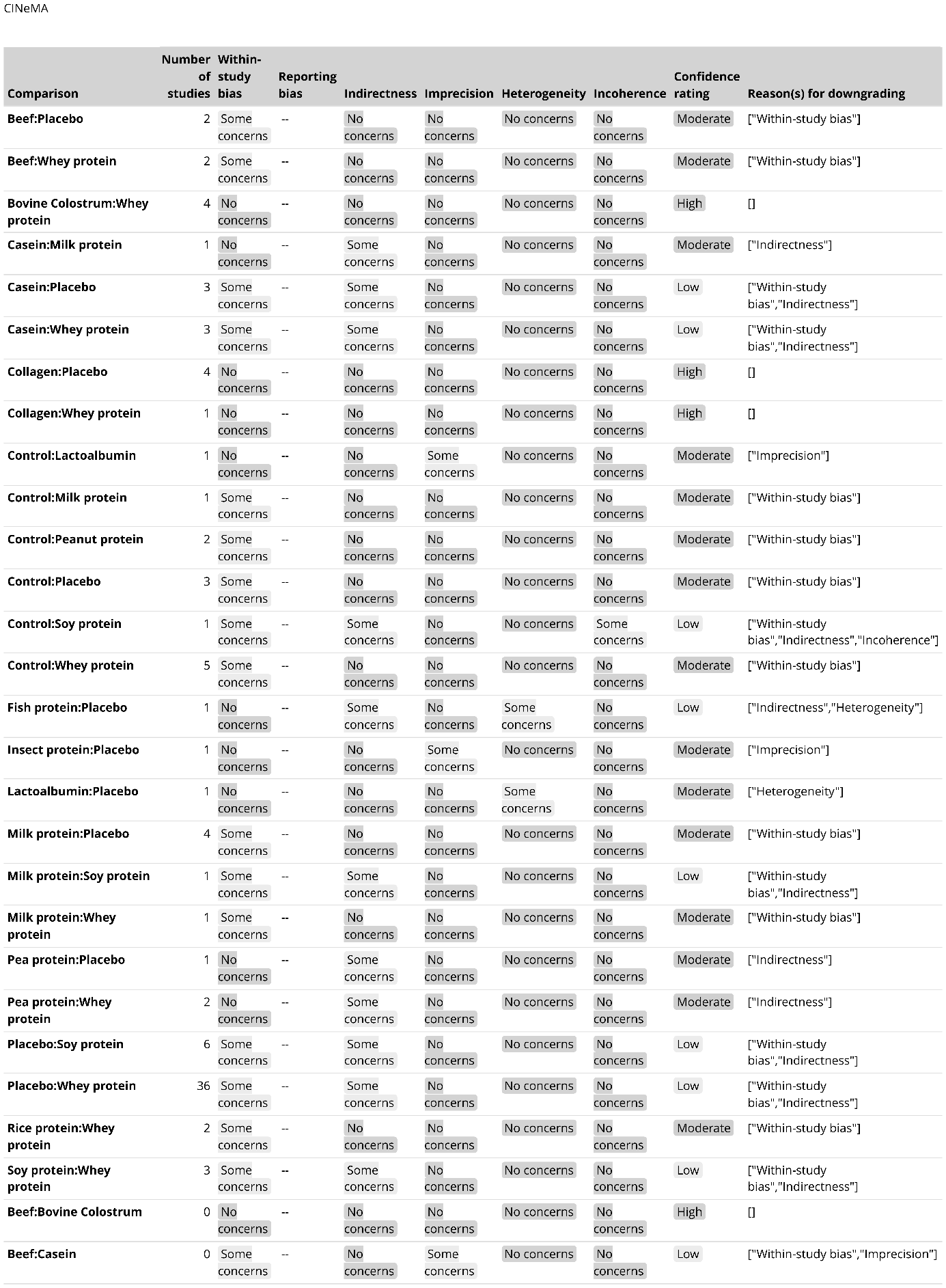


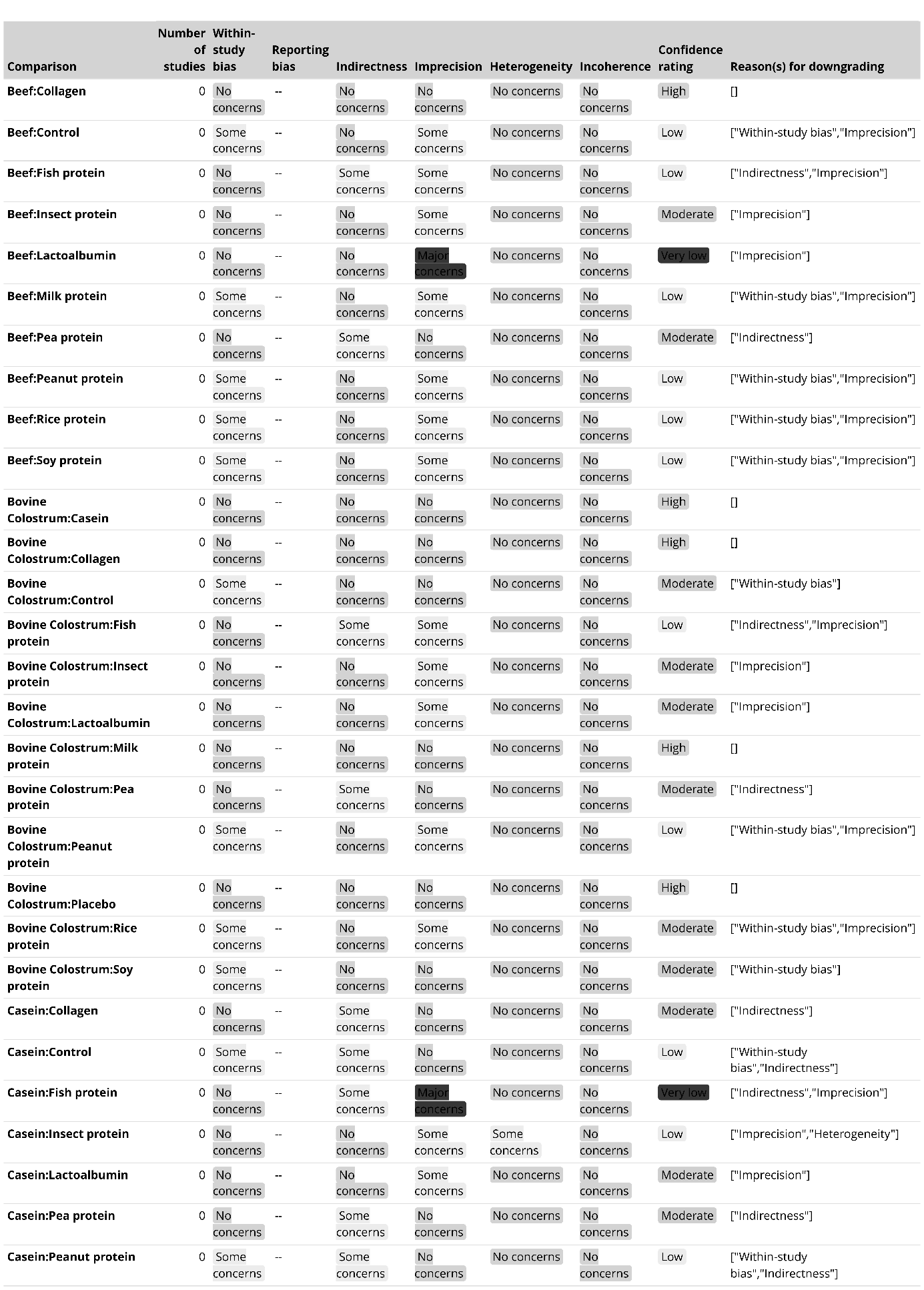


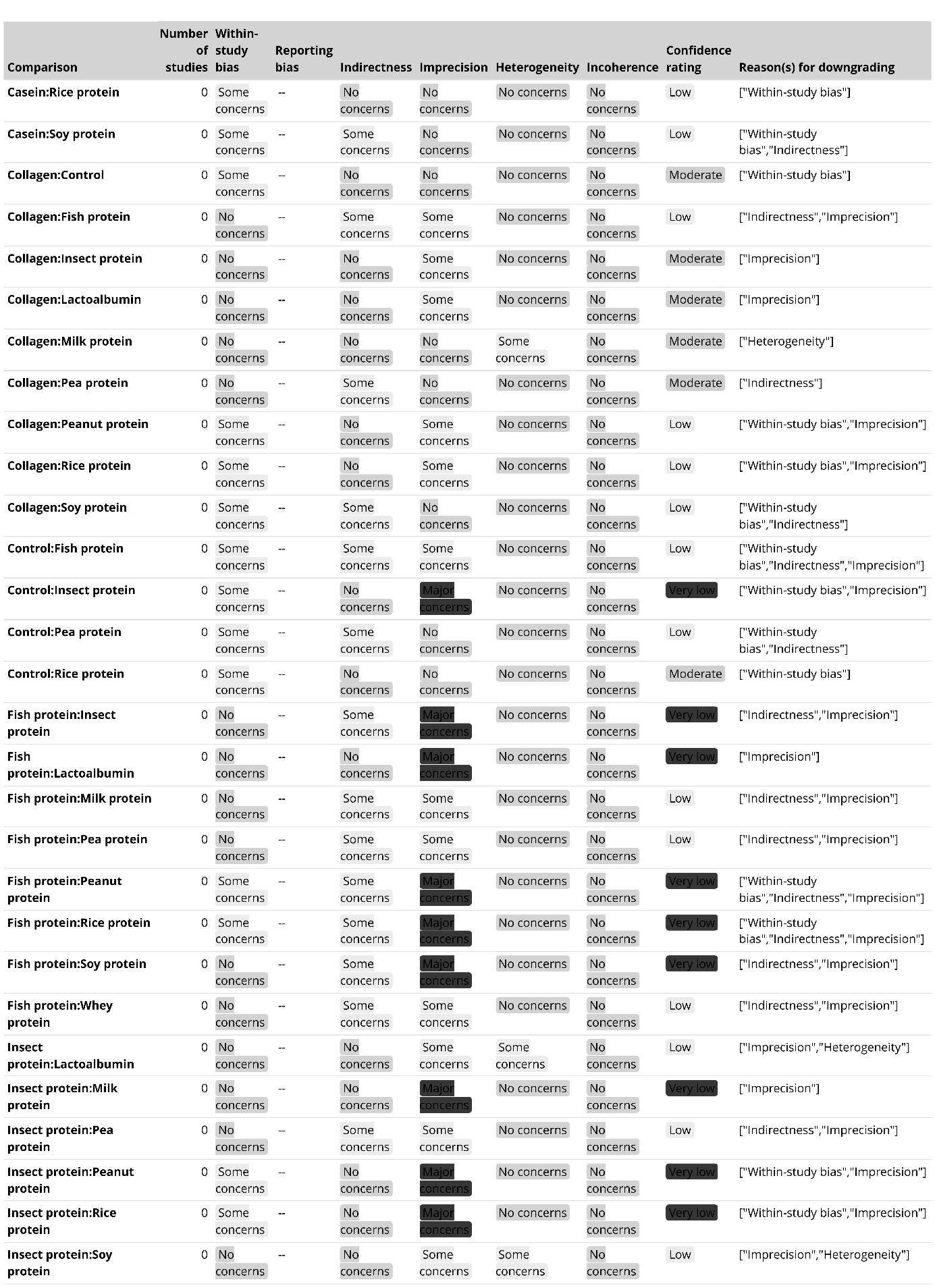


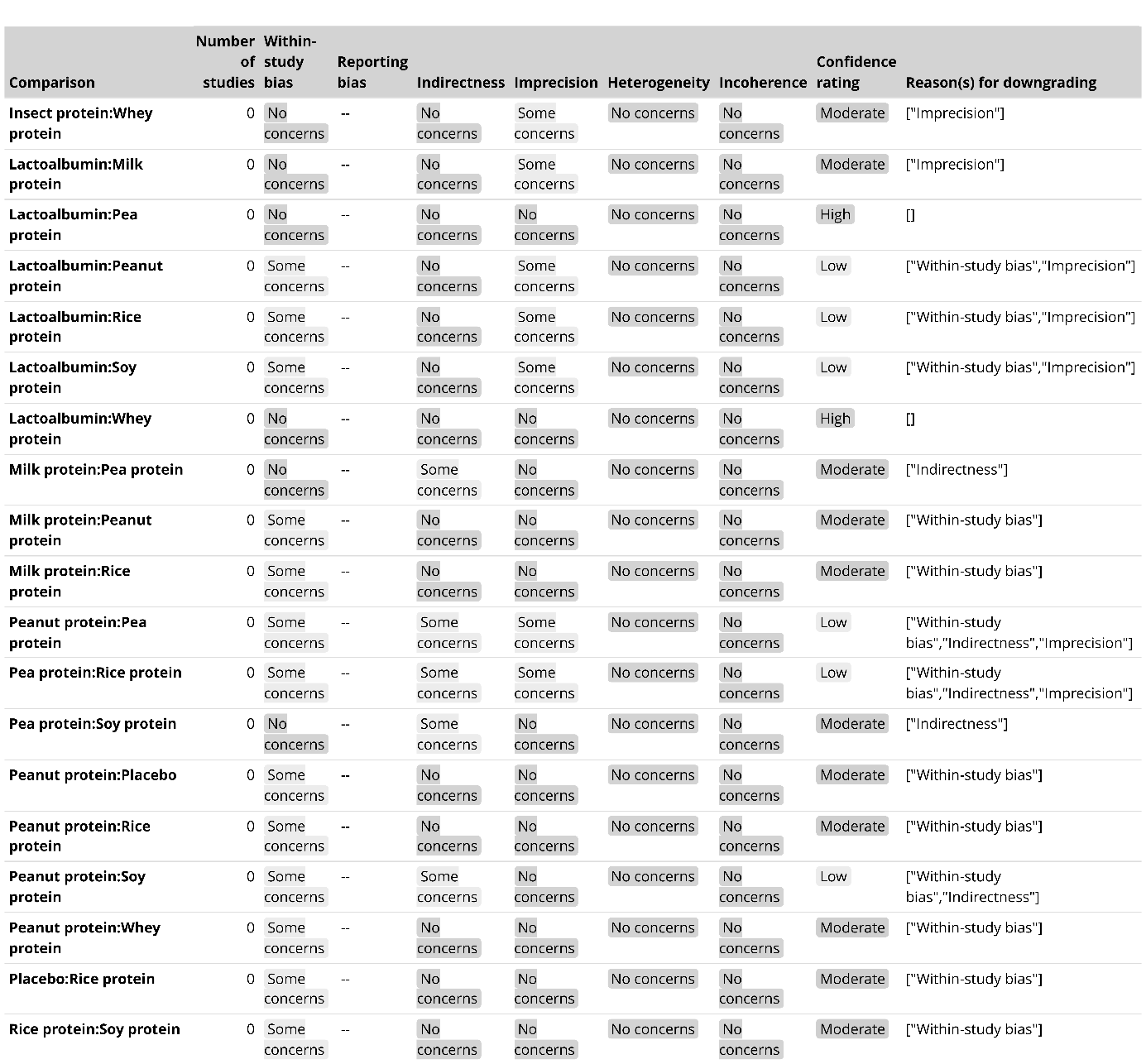


8.2 Figure S6: CINeMA for the Fat-Free Mass Outcome


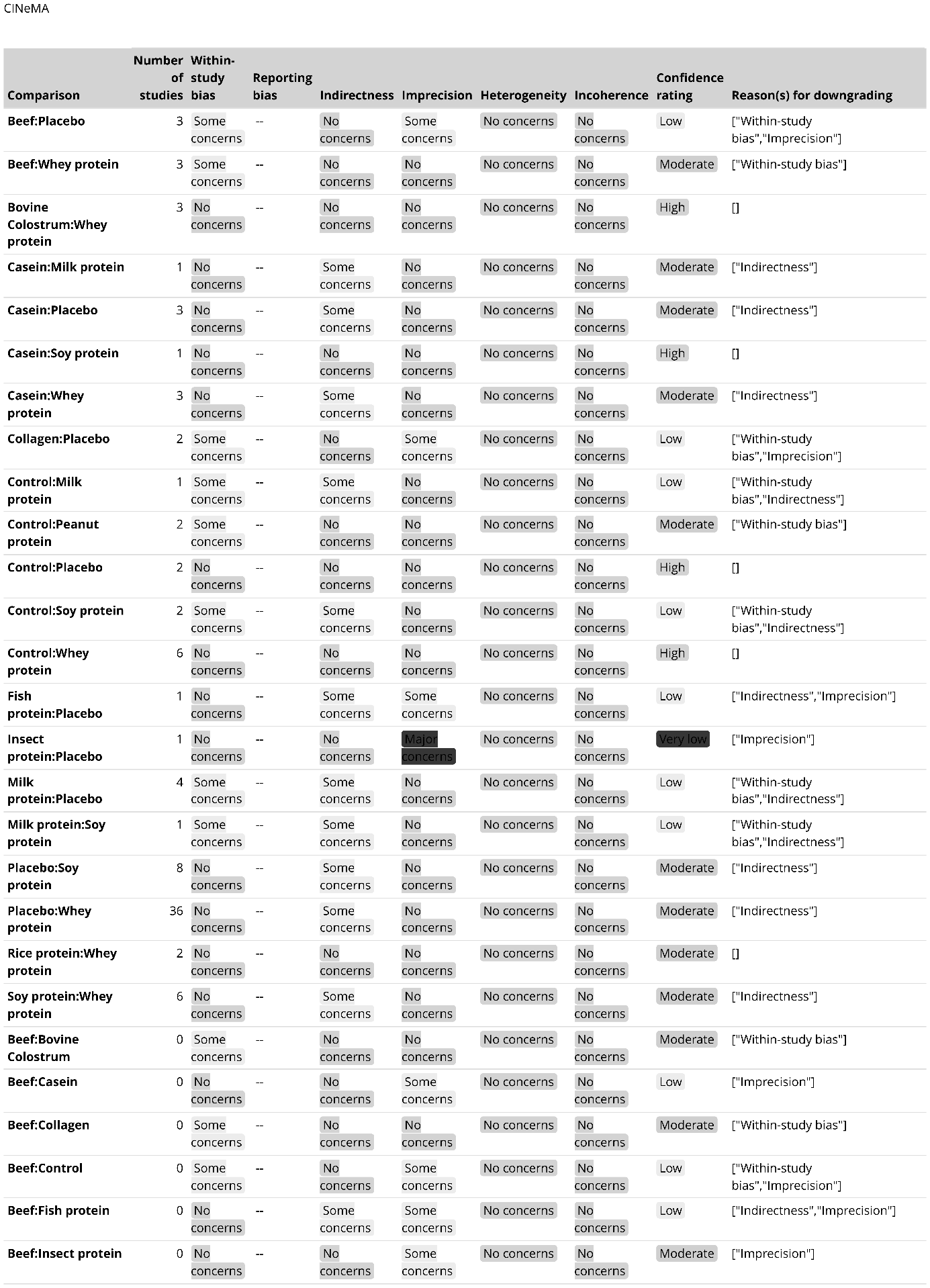


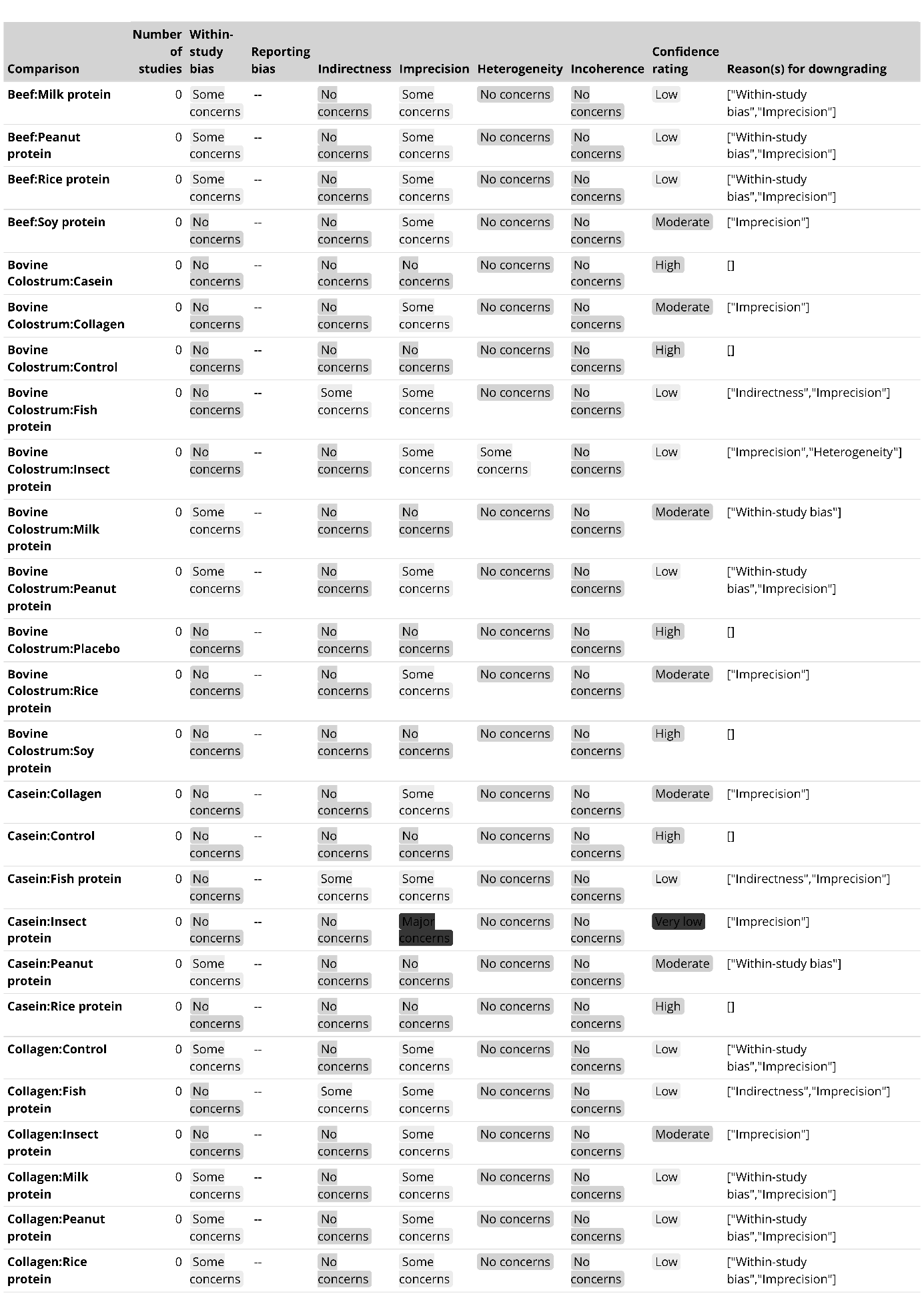


**
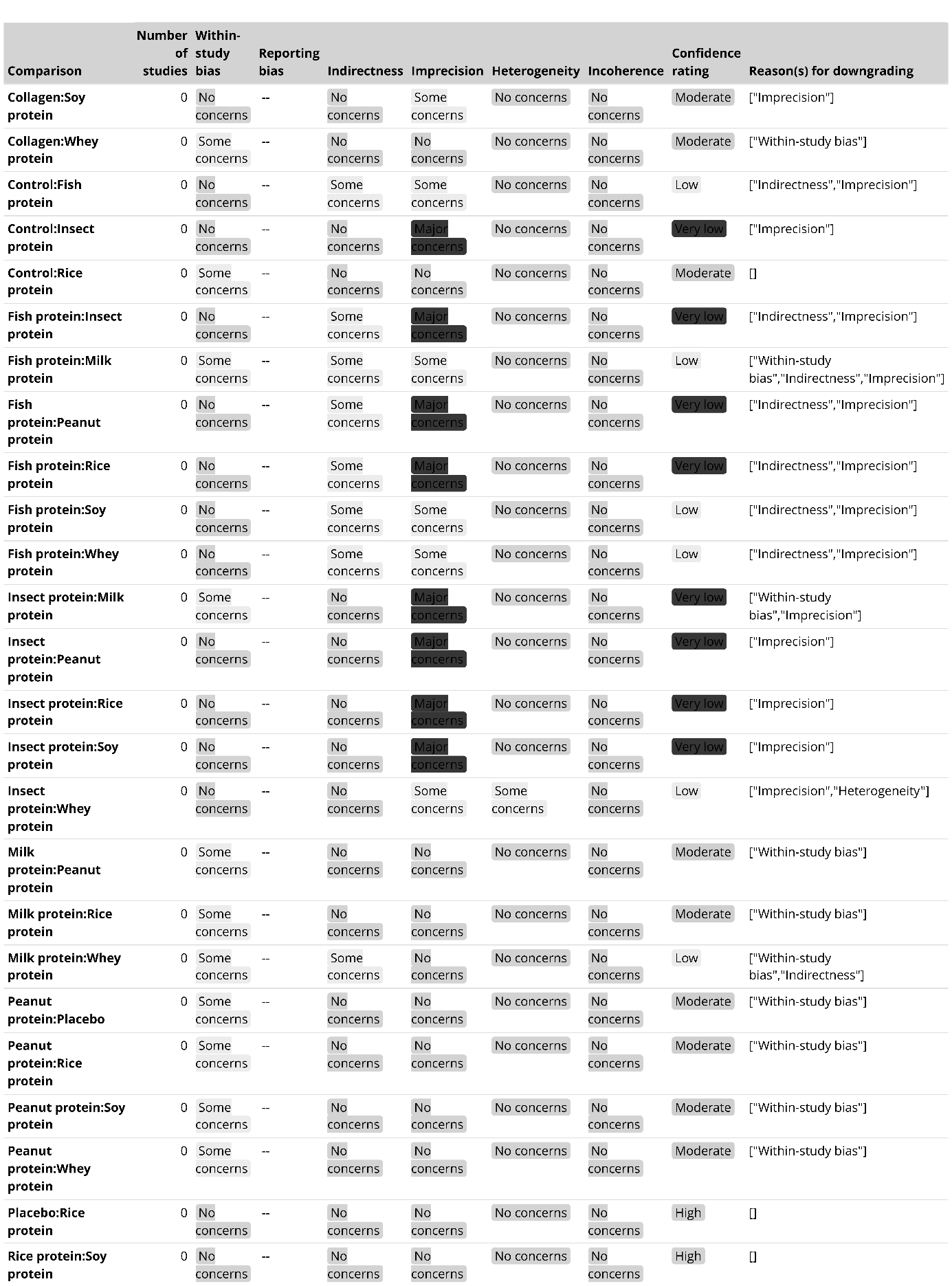
**

**Section 9 – Network Coherence Assessment**

9.1 Figure S7: Network Coherence for the Strength Outcome


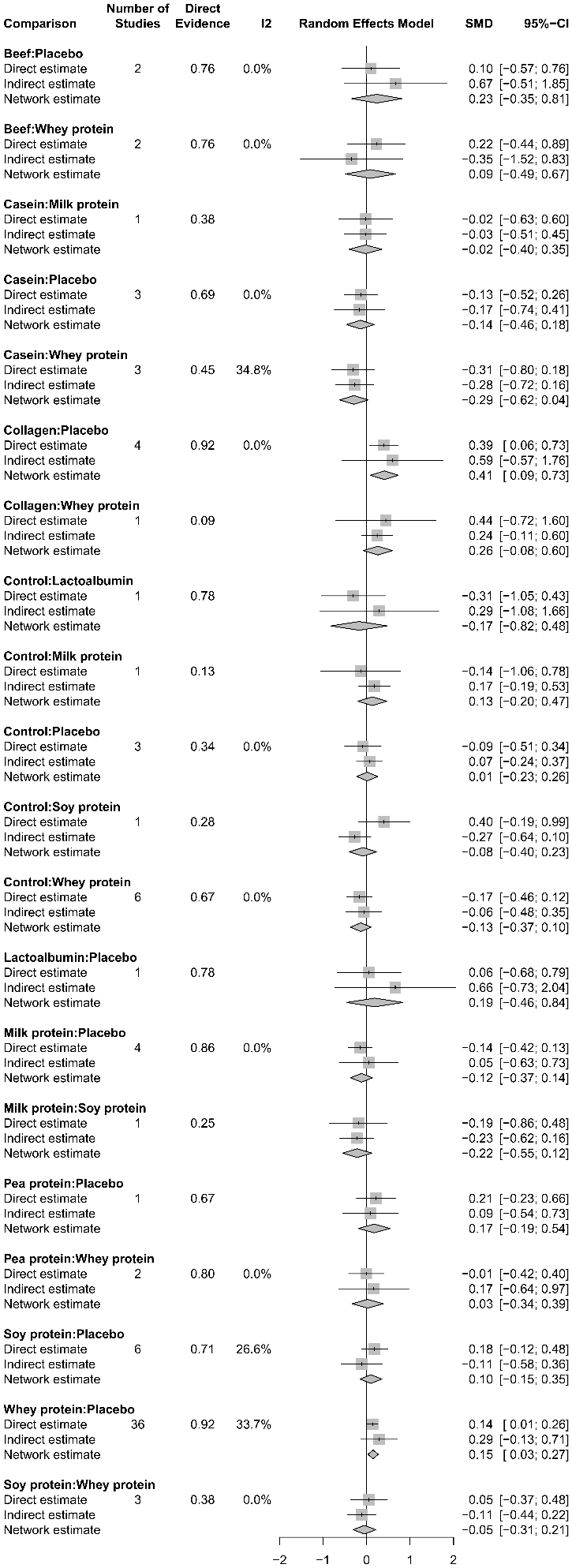


9.2 Figure S8: Network Coherence for the Fat-Free Mass Outcome


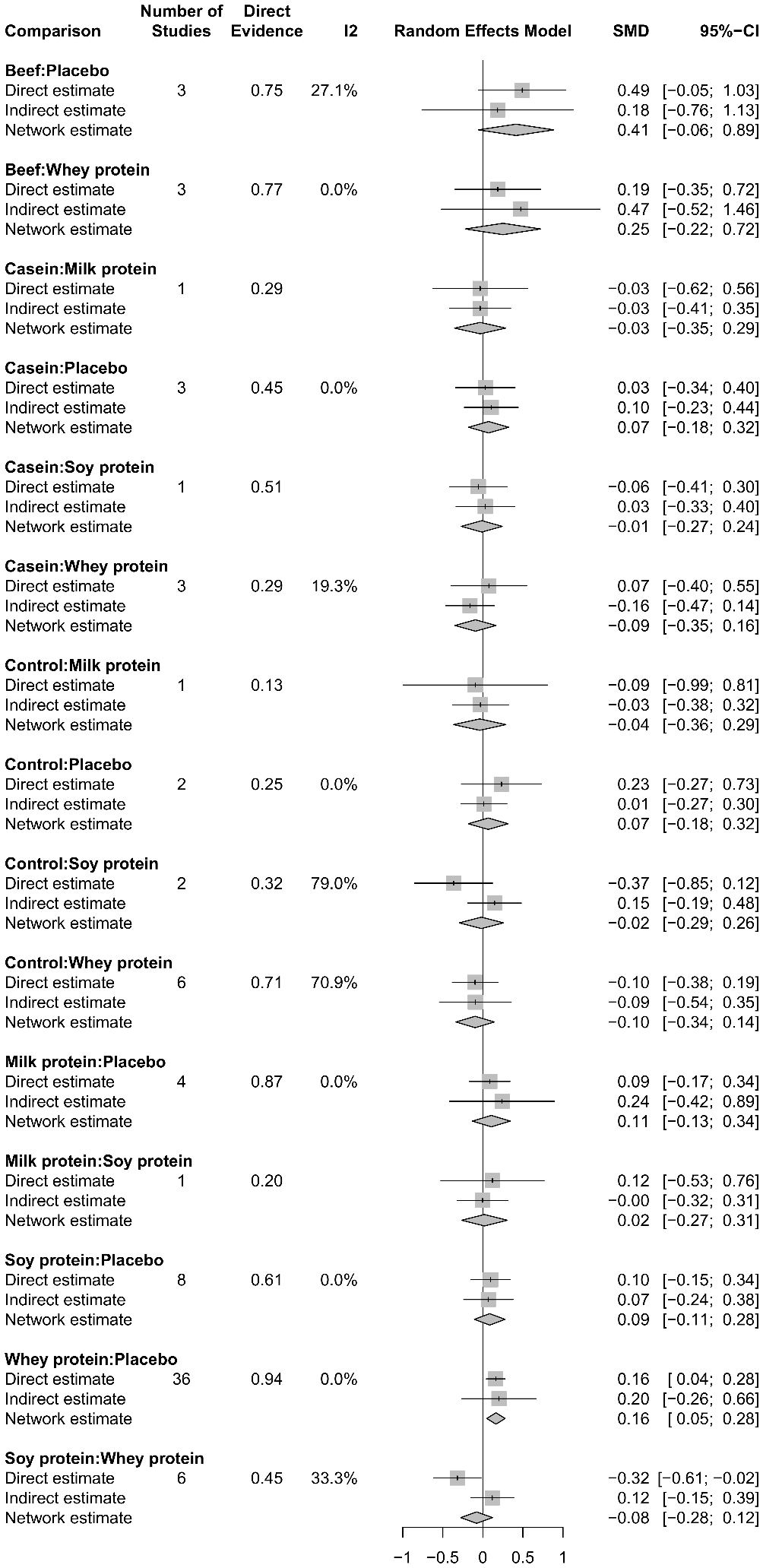


**Section 10 – Pairwise Meta-Analysis**

10.1 Figure S9: Pairwise Meta-Analysis for the Strength Outcome – Whey Protein vs Placebo


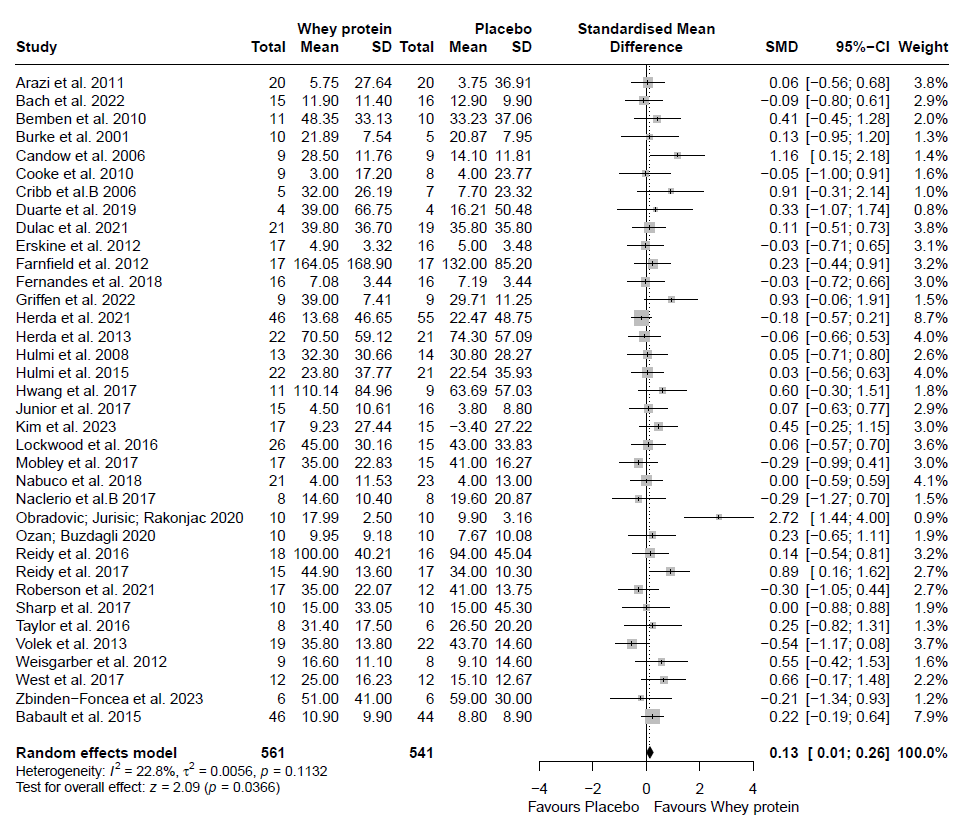


10.2 Figure S10: Pairwise Meta-Analysis for the Strength Outcome – Collagen vs Placebo


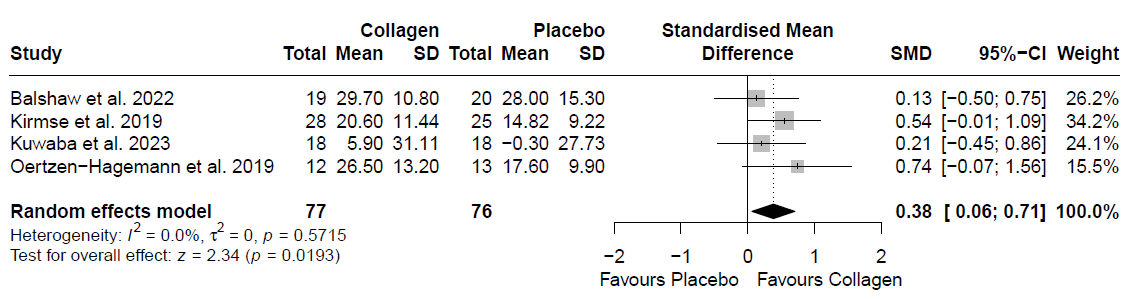


10.3 Figure S11: Pairwise Meta-Analysis for the Strength Outcome – Soy Protein vs Placebo*
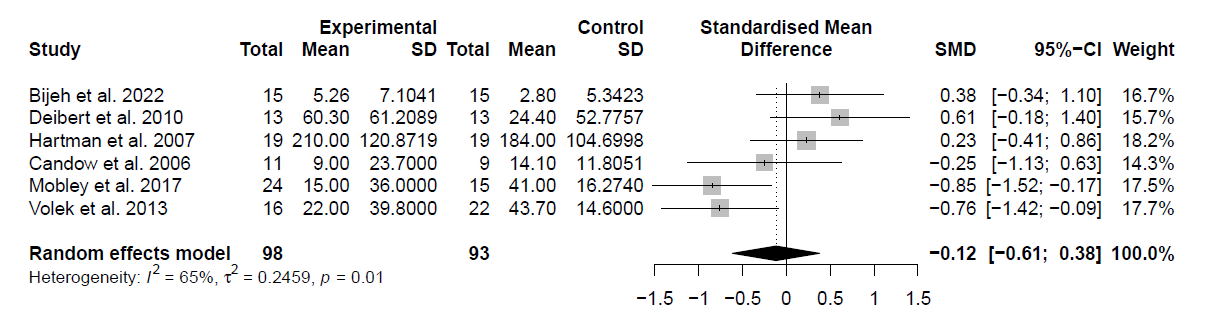
*

10.4 Figure S12: Pairwise Meta-Analysis for the Strength Outcome – Casein vs Placebo
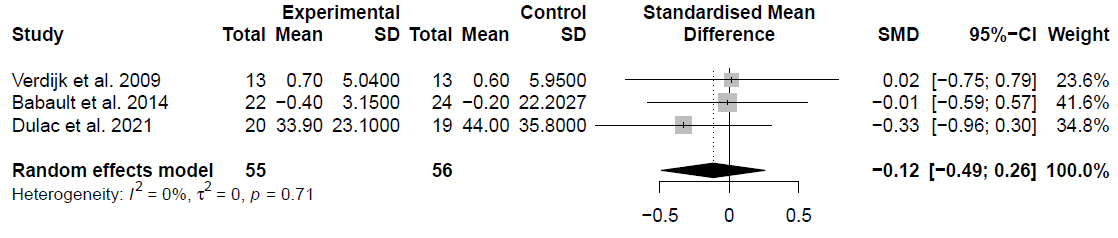


10.5 Figure S13: Pairwise Meta-Analysis for the Strength Outcome – Beef vs Placebo


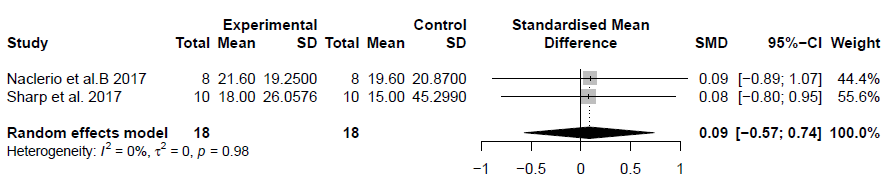


10.6 Figure S14: Pairwise Meta-Analysis for the Strength Outcome – Milk Protein vs Placebo
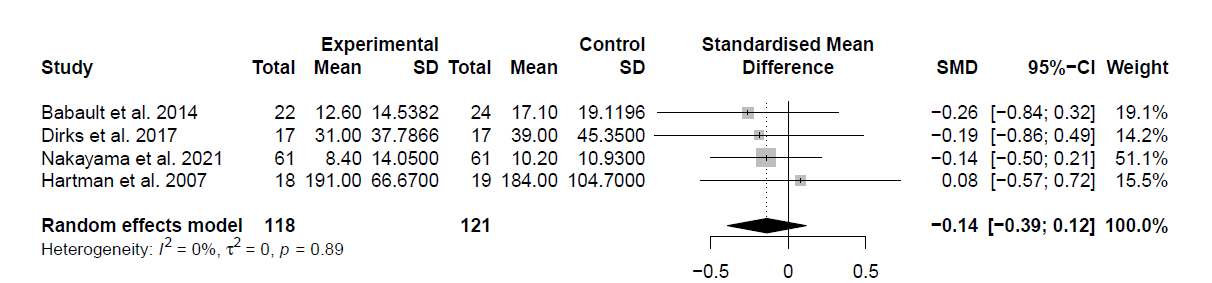


10.7 Figure S15: Pairwise Meta-Analysis for the Fat-Free Mass Outcome – Whey Protein vs Placebo
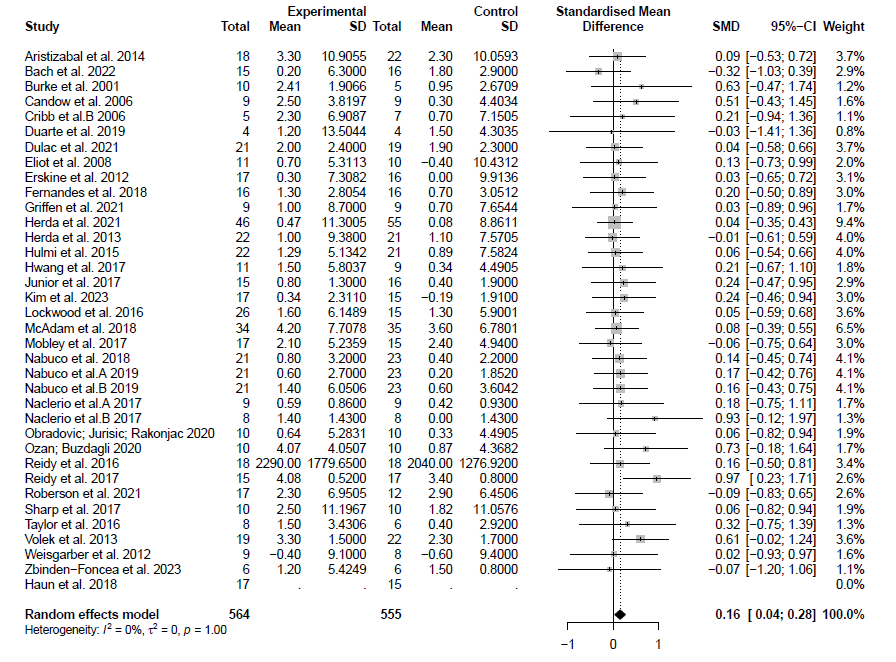


10.8 Figure S16: Pairwise Meta-Analysis for the Fat-Free Mass Outcome – Collagen vs Placebo
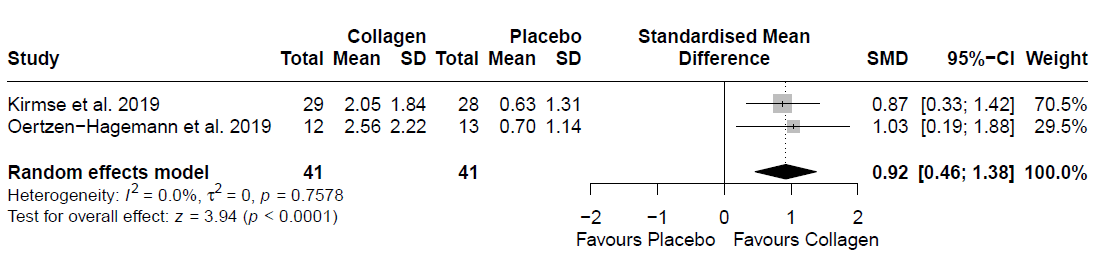


10.9 Figure S17: Pairwise Meta-Analysis for the Fat-Free Mass Outcome – Beef vs Placebo
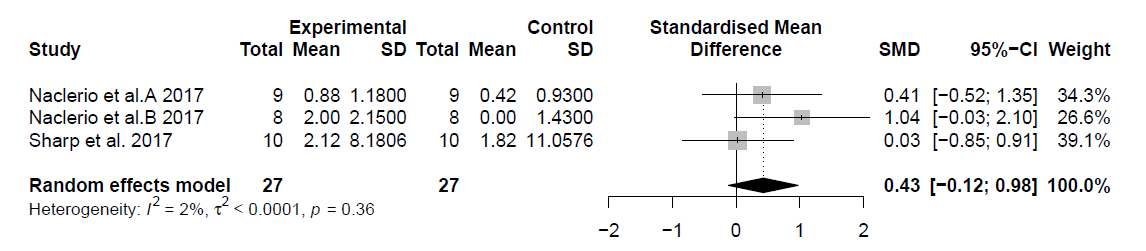


10.10 Figure S18: Pairwise Meta-Analysis for the Fat-Free Mass Outcome – Milk Protein vs Placebo
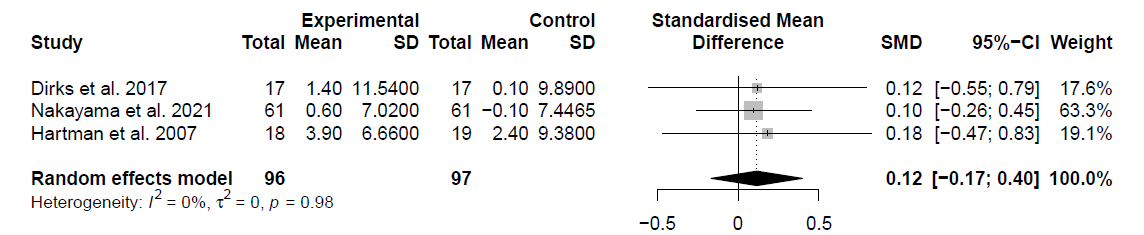


**Section 11. PRISMA NMA Checklist of Items to Include When Reporting A Systematic Review Involving a Network Meta-analysis**

| **Section/Topic** | **Item #** | **Checklist Item** | **Reported on Page #** |
| --- | --- | --- | --- |
| **TITLE** |  |  |  |
| Title | 1 | Identify the report as a systematic review *incorporating a network meta-analysis (or related form of meta-analysis).* | 1 |
| **ABSTRACT**  Structured summary    **INTRODUCTION** | 2 | Provide a structured summary including, as applicable:  **Background:** main objectives  **Methods:** data sources; study eligibility criteria, participants, and interventions; study appraisal; and *synthesis methods, such as network meta-analysis.*  **Results:** number of studies and participants identified; summary estimates with corresponding confidence/credible intervals; *treatment rankings may also be discussed. Authors may choose to summarize pairwise comparisons against a chosen treatment included in their analyses for brevity.* **Discussion/Conclusions:** limitations; conclusions and implications of findings.  **Other:** primary source of funding; systematic review registration number with registry name. | 2 |
| Rationale | 3 | Describe the rationale for the review in the context of what is already known*, including mention of why a network metaanalysis has been conducted.* | 3 |
| Objectives    **METHODS** | 4 | Provide an explicit statement of questions being addressed, with reference to participants, interventions, comparisons, outcomes, and study design (PICOS). | 4 |
| Protocol and registration | 5 | Indicate whether a review protocol exists and if and where it can be accessed (e.g., Web address); and, if available, provide registration information, including registration number. | 4 |
| Eligibility criteria 6 | | Specify study characteristics (e.g., PICOS, length of follow-up) and report characteristics (e.g., years considered, language, publication status) used as criteria for eligibility, giving rationale. *Clearly describe eligible treatments included in the treatment network, and note whether any have been clustered or merged into the same node (with justification).* | 4 |
| Information sources 7 | | Describe all information sources (e.g., databases with dates of coverage, contact with study authors to identify additional studies) in the search and date last searched. | 5 |
| Search 8 | | Present full electronic search strategy for at least one database, including any limits used, such that it could be repeated. | 5 |
| Study selection 9 | | State the process for selecting studies (i.e., screening, eligibility, included in systematic review, and, if applicable, included in the meta-analysis). | 5 |

| Data collection process | 10 | Describe method of data extraction from reports (e.g., piloted forms, independently, in duplicate) and any processes for obtaining and confirming data from investigators. | 6 |
| --- | --- | --- | --- |
| Data items | 11 | List and define all variables for which data were sought (e.g., PICOS, funding sources) and any assumptions and simplifications made. | 6 |
| **Geometry of the network** | **S1** | Describe methods used to explore the geometry of the treatment network under study and potential biases related to it. This should include how the evidence base has been graphically summarized for presentation, and what characteristics were compiled and used to describe the evidence base to readers. | 6 |
| Risk of bias within individual studies | 12 | Describe methods used for assessing risk of bias of individual studies (including specification of whether this was done at the study or outcome level), and how this information is to be used in any data synthesis. | 6 |
| Summary measures | 13 | State the principal summary measures (e.g., risk ratio, difference in means). *Also describe the use of additional summary measures assessed, such as treatment rankings and surface under the cumulative ranking curve (SUCRA) values, as well as modified approaches used to present summary findings from meta-analyses.* | 7 |
| Planned methods of analysis | 14 | Describe the methods of handling data and combining results of studies for each network meta-analysis. This should include, but not be limited to:   - *Handling of multi-arm trials;* - *Selection of variance structure;* - *Selection of prior distributions in Bayesian analyses; and* - *Assessment of model fit.* | 7 |
| **Assessment of Inconsistency** | **S2** | Describe the statistical methods used to evaluate the agreement of direct and indirect evidence in the treatment network(s) studied. Describe efforts taken to address its presence when found. | 8 |
| Risk of bias across studies | 15 | Specify any assessment of risk of bias that may affect the cumulative evidence (e.g., publication bias, selective reporting within studies). | 11 |
| Additional analyses | 16 | Describe methods of additional analyses if done, indicating which were pre-specified. This may include, but not be limited to, the following:   - Sensitivity or subgroup analyses; - Meta-regression analyses; - *Alternative formulations of the treatment network; and* - *Use of alternative prior distributions for Bayesian analyses (if applicable).* | 8 |

| **RESULTS†** |  |  |  |
| --- | --- | --- | --- |
| Study selection | 17 | Give numbers of studies screened, assessed for eligibility, and included in the review, with reasons for exclusions at each stage, ideally with a flow diagram. | 5 |
| **Presentation of network structure** | **S3** | Provide a network graph of the included studies to enable visualization of the geometry of the treatment network. | 10 |
| **Summary of network geometry** | **S4** | Provide a brief overview of characteristics of the treatment network. This may include commentary on the abundance of trials and randomized patients for the different interventions and pairwise comparisons in the network, gaps of evidence in the treatment network, and potential biases reflected by the network structure. | 10 |
| Study characteristics | 18 | For each study, present characteristics for which data were extracted (e.g., study size, PICOS, follow-up period) and provide the citations. | 10 |
| Risk of bias within studies | 19 | Present data on risk of bias of each study and, if available, any outcome level assessment. | 12 |
| Results of individual studies | 20 | For all outcomes considered (benefits or harms), present, for each study: 1) simple summary data for each intervention group, and 2) effect estimates and confidence intervals. *Modified approaches may be needed to deal with information from larger networks.* | 12-13-14 |
| Synthesis of results | 21 | Present results of each meta-analysis done, including confidence/credible intervals. *In larger networks, authors may focus on comparisons versus a particular comparator (e.g. placebo or standard care), with full findings presented in an appendix. League tables and forest plots may be considered to summarize pairwise comparisons.* If additional summary measures were explored (such as treatment rankings), these should also be presented. | 14 |
| **Exploration for inconsistency** | **S5** | Describe results from investigations of inconsistency. This may include such information as measures of model fit to compare consistency and inconsistency models, *P* values from statistical tests, or summary of inconsistency estimates from different parts of the treatment network. | 15-16 |
| Risk of bias across studies | 22 | Present results of any assessment of risk of bias across studies for the evidence base being studied. | 15 |
| Results of additional analyses    **DISCUSSION** | 23 | Give results of additional analyses, if done (e.g., sensitivity or subgroup analyses, meta-regression analyses*, alternative network geometries studied, alternative choice of prior distributions for Bayesian analyses,* and so forth). | 15 |
| Summary of evidence | 24 | Summarize the main findings, including the strength of evidence for each main outcome; consider their relevance to key groups (e.g., healthcare providers, users, and policymakers). | 16 |
| Limitations | 25 | Discuss limitations at study and outcome level (e.g., risk of bias), and at review level (e.g., incomplete retrieval of identified research, reporting bias). *Comment on the validity of the assumptions, such as transitivity and consistency. Comment* | 18 |
|  |  | *on any concerns regarding network geometry (e.g., avoidance of certain comparisons).* |  |
| Conclusions | 26 | Provide a general interpretation of the results in the context of other evidence, and implications for future research. | 19 |
| **FUNDING**  Funding | 27 | Describe sources of funding for the systematic review and other support (e.g., supply of data); role of funders for the systematic review. This should also include information regarding whether funding has been received from manufacturers of treatments in the network and/or whether some of the authors are content experts with professional conflicts of interest that could affect use of treatments in the network. | 19 |

PICOS = population, intervention, comparators, outcomes, study design.

* Text in italics indicateS wording specific to reporting of network meta-analyses that has been added to guidance from the PRISMA statement.

† Authors may wish to plan for use of appendices to present all relevant information in full detail for items in this section.

**Box. Terminology: Reviews With Networks of Multiple Treatments**

Different terms have been used to identify systematic reviews that incorporate a network of multiple treatment comparisons. A brief overview of common terms follows.

*Indirect treatment comparison:* Comparison of 2 interventions for which studies against a common comparator, such as placebo or a standard treatment, are available (i.e., indirect information). The direct treatment effects of each intervention against the common comparator (i.e., treatment effects from a comparison of interventions made within a study) may be used to estimate an indirect treatment comparison between the 2 interventions (**Appendix Figure 1, A**). An indirect treatment comparison (ITC) may also involve multiple links. For example, in **Appendix Figure 1, B**, treatments B and D may be compared indirectly on the basis of studies encompassing comparisons of B versus C, A versus C, and A versus D.

*Network meta-analysis* or *mixed treatment comparison*: These terms, which are often used interchangeably, refer to situations involving the simultaneous comparison of 3 or more interventions. Any network of treatments consisting of strictly unclosed loops can be thought of as a series of ITCs (**Appendix Figure 1, A and B**). In mixed treatment comparisons, both direct and indirect information is available to inform the effect size estimates for at least some of the comparisons; visually, this is shown by closed loops in a network graph (**Appendix Figure 1, C**). Closed loops are not required to be present for every comparison under study. "Network meta-analysis" is an inclusive term that incorporates the scenarios of both indirect and mixed treatment comparisons.

*Network geometry evaluation:* The description of characteristics of the network of interventions, which may include use of numerical summary statistics. This does not involve quantitative synthesis to compare treatments. This evaluation describes the current evidence available for the competing interventions to identify gaps and potential bias. Network geometry is described further in **Appendix Box 4**.

| **Appendix Box 1. The Assumption of Transitivity for Network Meta-Analysis** Methods for indirect treatment comparisons and network meta-analysis enable learning about the relative treatment effects of, for example, treatments A and B through use of studies where these interventions are compared against a common therapy, C.    When planning a network meta-analysis, it is important to assess patient and study characteristics across the studies that compare pairs of treatments. These characteristics are commonly referred to as *effect modifiers* and include traits such as average patient age, gender distribution, disease severity, and a wide range of other plausible features.    For network meta-analysis to produce valid results, it is important that the distribution of effect modifiers is similar, for example, across studies of A versus B and A versus C. This balance increases the plausibility of reliable findings from an indirect comparison of B versus C through the common comparator A. When this balance is present, the assumption of transitivity can be judged to hold.    Authors of network meta-analyses should present systematic (and even tabulated) information regarding patient and study characteristics whenever available. This information helps readers to empirically evaluate the validity of the assumption of transitivity by reviewing the distribution of potential effect modifiers across trials. |
| --- |

| **Appendix Box 2. Differences in Approach to Fitting Network Meta-Analyses** Network meta-analysis can be performed within either a frequentist or a Bayesian framework. Frequentist and Bayesian approaches to statistics differ in their definitions of probability. Thus far, the majority of published network meta-analyses have used a Bayesian approach.    Bayesian analyses return the posterior probability distribution of all the model parameters given the data and prior beliefs (e.g., from external information) about the values of the parameters. They fully encapsulate the uncertainty in the parameter of interest and thus can make direct probability statements about these parameters (e.g., the probability that one intervention is superior to another).    Frequentist analyses calculate the probability that the observed data would have occurred under their sampling distribution for hypothesized values of the parameters. This approach to parameter estimation is more indirect than the Bayesian approach.    Bayesian methods have been criticized for their perceived complexity and the potential for subjectivity to be introduced by choice of a prior distribution that may affect study findings. Others argue that explicit use of a prior distribution makes transparent how individuals can interpret the same data differently. Despite these challenges, Bayesian methods offer considerable flexibility for statistical modeling. In-depth introductions to Bayesian methods and discussion of these and other issues can be found elsewhere. |
| --- |

| **Appendix Box 3. Network Meta-Analysis and Assessment of Consistency** Network meta-analysis often involves the combination of direct and indirect evidence. In the simplest case, we wish to compare treatments A and B and have 2 sources of information: direct evidence via studies comparing A versus B, and indirect evidence via groups of studies comparing A and B with a common intervention, C. Together, this evidence forms a closed loop, ABC.    Direct and indirect evidence for a comparison of interventions should be combined only when their findings are similar in magnitude and interpretation. For example, for a comparison of mortality rates between A and B, an odds ratio determined from studies of A versus B should be similar to the odds ratio comparing A versus B estimated indirectly based on studies of A versus C and B versus C. This assumption of comparability of direct and indirect evidence is referred to as *consistency* of treatment effects.    When a treatment network contains a closed loop of interventions, it is possible to examine statistically whether there is agreement between the direct and indirect estimates of intervention effect.    Different methods to evaluate potential differences in relative treatment effects estimated by direct and indirect comparisons are grouped as *local approaches* and *global approaches.* Local approaches (e.g., the Bucher method or the node-splitting method) assess the presence of inconsistency for a particular pairwise comparison in the network, whereas global approaches (e.g., inconsistency models, *I*^2^ measure for inconsistency) consider the potential for inconsistency in the network as a whole.    Tests for inconsistency can have limited power to detect a true difference between direct and indirect evidence. When multiple loops are being tested for inconsistency, one or a few may show inconsistency simply by chance. Further discussions of consistency and related concepts are available elsewhere.  Inconsistency in a treatment network can indicate lack of transitivity (see **Appendix Box 1**). |
| --- |

| **Appendix Box 4. Network Geometry and Considerations for Bias**  The term *network geometry* is used to refer to the architecture of the treatment comparisons that have been made for the condition under study. This includes what treatments are involved in the comparisons in a network, in what abundance they are present, the respective numbers of patients randomly assigned to each treatment, and whether particular treatments and comparisons may have been preferred or avoided.    Networks may take on different shapes. Poorly connected networks depend extensively on indirect comparisons. Meta-analyses of such networks may be less reliable than those from networks where most treatments have been compared against each other.    Qualitative description of network geometry should be provided and accompanied by a network graph. Quantitative metrics assessing features of network geometry, such as *diversity* (related to the number of treatments assessed and the balance of evidence among them), *co-occurrence* (related to whether comparisons between certain treatments are more or less common), and *homophily* (related to the extent of comparisons between treatments in the same class versus competing classes), can also be mentioned.    Although common, established steps for reviewing network geometry do not yet exist, however examples of in-depth evaluations have been described related to treatments for tropical diseases and basal cell carcinoma and may be of interest to readers. An example based on 75 trials of treatments for pulmonary arterial hypertension (**Appendix Figure 3**) suggests that head-to-head studies of active therapies may prove useful to further strengthen confidence in interpretation of summary estimates of treatment comparisons. |
| --- |

**Appendix Box 5. Probabilities and Rankings in Network Meta-Analysis** Systematic reviews incorporating network meta-analyses can provide information about the hierarchy of competing interventions in terms of treatment rankings.

The term *treatment ranking probabilities* refers to the probabilities estimated for each treatment in a network of achieving a particular placement in an ordering of treatment effects from best to worst. A network of 10 treatments provides a total of 100 ranking probabilities—that is, for each intervention, the chance of being ranked first, second, third, fourth, fifth, and so forth).

Several techniques are feasible to summarize relative rankings, and include graphical tools as well as different approaches for estimating ranking probabilities. **Appendix Figure 6** shows 2 approaches to presenting such information, on the basis of a comparison of adjuvant interventions for resected pancreatic adenocarcinoma.

Robust reporting of rankings also includes specifying median ranks with uncertainty intervals, cumulative probability curves, and the surface under the cumulative ranking (SUCRA) curve.

Rankings can be reported along with corresponding estimates of pairwise comparisons between interventions. Rankings should be reported with probability estimates to minimize misinterpretation from focusing too much on the most likely rank.

Rankings may exaggerate small differences in relative effects, especially if they are based on limited information. An objective assessment of the strength of information in the network and the magnitude of absolute benefits should accompany rankings to minimize potential biases.

**Appendix Figure 1A-1C**


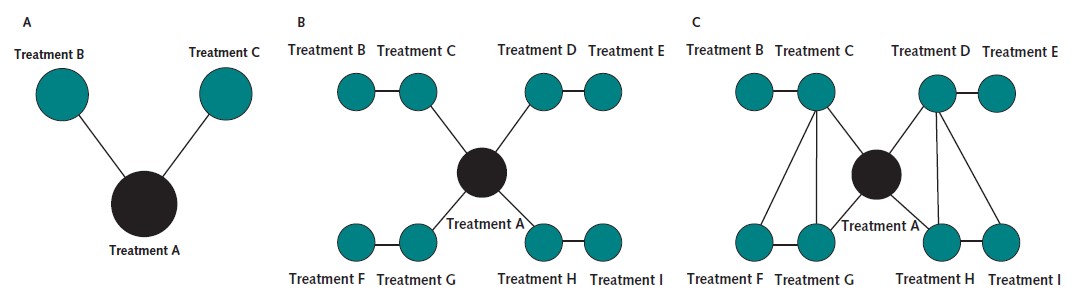


**Appendix Figure 3**


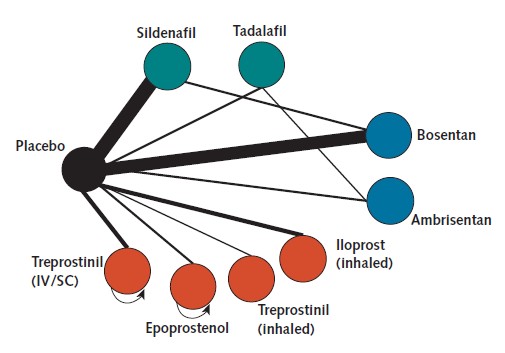


**Appendix Figure 6**


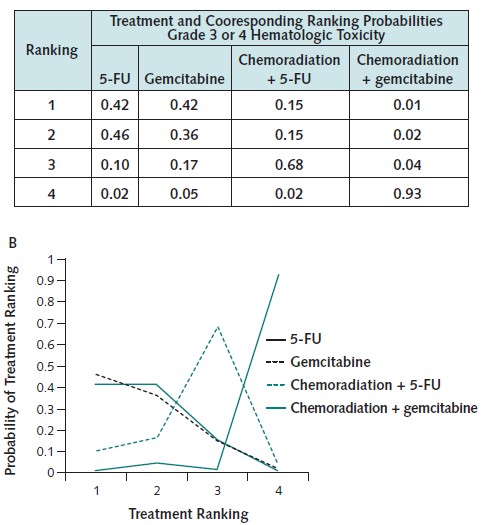

Supplement: Supplementary file 1 — Supporting Information Additional supporting information can be found online in the Supporting Information section. [file TSM2-2026-5557511-s001.docx]
